# Happiness feels light and sadness feels heavy: introducing valence-related bodily sensation maps of emotions

**DOI:** 10.1007/s00426-022-01661-3

**Published:** 2022-02-28

**Authors:** Matthias Hartmann, Bigna Lenggenhager, Kurt Stocker

**Affiliations:** 1 Faculty of Psychology, UniDistance Suisse, Ueberlandstrasse 12, 3900 Brig, Switzerland; 2 Department of Psychology, University of Bern, Bern, Switzerland; 3 Department of Psychology, University of Zurich, Zurich, Switzerland; 4 Department of Humanities, Social and Political Sciences, Chair of Cognitive Science, ETH Swiss Federal Institute of Technology Zurich, Zurich, Switzerland; 5 Psychopharmacology Research, Division of Clinical Pharmacology and Toxicology, Department of Clinical Research, University Hospital Basel, University of Basel, Basel, Switzerland

## Abstract

Bodily sensation mapping (BSM) is a recently developed self-report tool for the assessment of emotions in which people draw their sensations of *activation* in a body silhouette. Following the circumplex model of affect, activity *and valence* are the underling dimensions of every emotional experience. The aim of this study was to introduce the neglected valence dimension in BSM. We found that participants systematically report valence-related sensations of bodily lightness for positive emotions (happiness, love, pride), and sensations of bodily heaviness in response to negative emotions (e.g., anger, fear, sadness, depression) with specific body topography (Experiment 1). Further experiments showed that both computers (using a machine learning approach) and humans recognize emotions better when classification is based on the combined activity- and valence-related BSMs compared to either type of BSM alone (Experiments 2 and 3), suggesting that both types of bodily sensations reflect distinct parts of emotion knowledge. Importantly, participants found it clearer to indicate their bodily sensations induced by sadness and depression in terms of bodily weight than bodily activity (Experiment 2 and 4), suggesting that the added value of valence-related BSMs is particularly relevant for the assessment of emotions at the negative end of the valence spectrum.

**Supplementary Information:**

The online version contains supplementary material available at 10.1007/s00426-022-01661-3.

## Introduction

Emotions play a central role in everyday life: they guide our behavior toward or away from positive or negative incentives, and they interact with information processing and evaluation when making decisions (Buck, 1985; Forgas, 1994; Frijda, 1987; Schwarz, 1990). Despite this relevance and a long history of research, no consensus about the definition, function, underlying physiological processes, and measurement of emotions has been reached (e.g., Ekman, 2016; Gainotti, 2020; Izard, 2013). This is partly due to the fact that emotions are multifaceted phenomena involving physiological responses across the whole body, and changes in subjective feelings, cognition, and behavior (Damasio, 2004; Levenson, 2019; Scherer, 2000).

One of the major controversies is related to the question of whether emotions are best organized in terms of dimensions or in terms of discrete categories (Barrett & Wager, 2006; Kragel & LaBar, 2016; Posner et al., 2005). Discrete models of emotions assume that a limited number of universal and innate basic emotions have been developed during the course of evolution—such as fear, anger, disgust, sadness, and happiness—which subserve adaptational strategies (Ekman, 1992, 1999). Distinct emotion models typically build on the assumption that each emotion has its own specific eliciting condition and its own specific neural, physiological, expressive, and behavioral reaction pattern (Ekman, 1992; Izard, 1991; Tomkins, 1984). Scientific evidence for this assumption is mixed and the matter remains under debate (Barrett & Wager, 2006; Cacioppo et al., 2000; Panksepp, 2007).

Instead of specific mechanisms for each emotion, dimensional models assume that all affective states can be understood as varying degrees of common neurophysiological or “psychological” dimensions. Most prominently, the two-dimensional circumplex model of affect proposed that each emotional state can be described in terms of the level of arousal/activity, and in terms of valence (pleasantness, unpleasantness) (Russel, 1980). Although additional dimensions such as attention-rejection, dominance-submissiveness, power, or intensity have also been proposed (Bradley & Lang, 1994; Mehrabian, 1996; Osgood et al., 1957; Schlosberg, 1954; Wundt, 1905), two-dimensional bipolar activation/valence models remain the most influential ones (Bliss-Moreau et al., 2020; Posner et al., 2005). For example, the arousal and valence dimension accounts for the major proportion of variance in judged similarities among emotion words, suggesting that these two dimensions are the basic properties of the cognitive representation of different emotional states (Bush, 1973; Russel, 1980).

Dimensional models typically build on the assumption that each organism possesses basic approach and avoidance tendencies, and that different emotions emerged from these basic responses in combination with the cognitive interpretation of the situation—valence determines the direction (approach vs. avoidance) and arousal determines the intensity of such basic responses (e.g., Cacioppo & Berntson, 1994; Lang et al., 1998; Russell, 2003). Recent neuroimaging studies confirmed the existence of distinct neuronal networks subserving the valence and arousal dimension of emotions (Colibazzi et al., 2010; Posner et al., 2009), but also the dimensional approach remains debated (e.g., Kragel & LaBar, 2016).

While the discrete and dimensional approaches to emotions are often contrasted against each other (e.g., Adolphs, 2017; Barrett, 2006; Izard, 2007), some authors argued that the issue about the format of representation will most likely never be resolved and that both approaches make valid contributions in understanding emotions (Ekman, 2016; Harmon-Jones et al., 2017). Such an integrative view might also advance the measurement of emotions, which often has not progressed along with theory (Harmon-Jones et al., 2016). Emotions are typically measured using physiological/behavioral and/or self-report measurements. Research driven by distinct models typically focuses on physiological/behavioral responses from facial muscles or from the autonomic nervous system (Ekman & Friesen, 1971; Ekman et al., 1983; Gomez et al., 2005; Konishi et al., 2019; Levenson, 2014; Nardelli et al., 2015; Siegel et al., 2018). Limitations of this line of research are that it is laborious, that often only one specific physiological response is measured, and that the component of subjective feeling is often neglected. Research driven by dimensional models typically focus on self-report measurements (Harmon-Jones et al., 2016). For example, one of the most widely used self-report measurements is the Positive and Negative Affect Schedule (PANAS; Watson et al., 1988), asking participants to indicate to what extent positive and negative emotional words describe their feelings. Other typical self-report measurements require to choose a graphical (e.g., faces, manikins) or numeric representation of emotions on valence and arousal (and sometimes other) dimensions (e.g., Bradley & Lang, 1994; Broekens & Brinkman, 2013; Mehrabian, 1995).

These well-established self-report measurements come along with some limitations. For example, the PANAS has been criticized (among others) for not measuring positive and negative affect directly but rather positive and negative activation (e.g., alert, active, attentive) (Harmon-Jones et al., 2016). This can lead to misleading results when negatively valenced activation is involved, such as an increase of the positive affect score for anger (Harmon-Jones et al., 2009). Such limitations have partly been overcome by assessing emotional states on a two-dimensional valence/arousal affect grid (e.g., Russel, 1980). Nevertheless, it remains a challenge for participants to express subjective feelings of arousal and valence by means of a position on a rather abstract scale, and for researchers and therapists to accurately classify emotions based on these spatial points (Harmon-Jones et al., 2016). Various other self-report measurements have been developed with a specific focus on a single emotion category (e.g., Haidt et al., 1994; Lyubomirsky & Lepper, 1999; Smith et al., 1999; Spielberger, 2010; Tracy & Robins, 2007) or on the clinical context (e.g., McNair et al., 1971; Zuckerman et al., 1965). Although more comprehensive self-report measurements have been developed in the past (e.g., Harmon-Jones et al., 2016; Plutchik, 2001), there is room for further measurements that take aspects of both the dimensional and discrete models of emotion into account, apply for a wide-range of emotions and are easily applicable.

## Bodily sensation mapping

One such novel, innovative tool for the assessment of emotions is “Bodily sensation mapping” (Nummenmaa et al., 2014). Instead of reporting feelings on a scale, participants *draw* their feelings in the format of bodily sensations (i.e., felt changes in bodily activity in response to emotions) in a whole-body silhouette. These drawings result in bodily sensation maps (BSMs), showing distinct activation patterns across the whole body for different emotional states (Nummenmaa et al., 2014). The same emotion-specific BSM patterns were found across cultures and also across different emotion induction techniques such as self-displacement by emotion words, and emotion induction by emotional videos (Nummenmaa et al., 2014; Volynets et al., 2019).

Bodily sensation mapping combines important elements from both the dimensional and discrete approach to emotions. It is basically a dimensional approach, since participants can indicate the degree of sensation on the activation dimension (e.g., larger and more intense painting reflects more widespread and intense sensations of bodily activation), and it allows doing this in a body-specific, topographical way. Bodily sensation mapping therefore accounts for the fact that emotions are associated with a variety of emotion-specific physiological changes and are consequently “felt” across the entire body (e.g., Barrett et al., 2007; Damasio & Carvalho, 2013; James, 1884; Kim & André, 2008). Given that BSMs rely on self-report, it remains open to what extent BSM patterns truly reflect physiological changes. Nummenmaa et al. (2014) argued that BSMs may not necessarily reflect conscious awareness of a *specific* physiological change but rather *net sensations* from different physiological systems (skeletomuscular and visceral sensations, effects of autonomic nervous system). In line with this idea, BSM patterns correspond with the major changes in physiological functions associated with the different emotions (Izard, 1991), such as an increased activity in the upper chest area for most basic emotions, likely corresponding to changes in breathing and heart rate (Levenson, 2003), or increased activity in the upper limbs for approach-oriented emotions such as anger and happiness (Nummenmaa et al., 2014). Moreover, it has been shown that individuals with higher interoceptive accuracy created more intense and topographically more specific BSM patterns following exposure to emotional stimuli (Jung et al., 2017), supporting the view that BSMs may reflect interoceptively experienced physiological changes.

Regardless of the exact mechanisms underlying BSMs, they have important theoretical and practical implications. On the theoretical level, the fact that the reported bodily sensations are topographically distinct suggests that specific bodily sensations are a crucial part of emotion knowledge (Nummenmaa et al., 2014). In line with the embodied view of cognition and emotion, changes in bodily states constitute the body’s registering of how it is being altered by its engagement with the environment, and we feel an emotional state whenever we become aware of those changes in our bodily state (e.g., Foglia & Wilson, 2013; Hufendiek, 2015; Johnson, 2015). The distinct topographical representations of bodily sensations may play an important role for becoming aware about one’s emotional state and for the emergence of one’s emotional feelings (Nummenmaa et al., 2014), which eventually provide meaning to a situation (Johnson, 2015).

On the practical level, BSMs may be a useful tool to improve the specificity and efficiency of communicating and visualizing emotional feelings. People typically have difficulty in assessing, discerning, and describing their own emotions (Becker‐Weidman & Hughes, 2008; Saarni, 1999). Instead of describing their emotional feelings verbally or transferring their feelings into an abstract value on a scale, people can just “paint” a BSM to represent how their body feels. Nummenmaa et al. (2014) proposed BSMs as “somatic markers” for emotional disorders (although we think that the term “bodily sensation markers” is more appropriate, since no somatic measurements are used). BSM is also a promising tool for emotion detection in the context of user experience. A recently developed smartphone application showed that using a BSM approach leads to better classification of the user’s emotion when compared to the classical affect grid approach in which participants provide a single (i.e., body-unspecific) arousal and valence value (García-Magariño et al., 2018).

However, before the potential of BSMs can be exploited and its theoretical and practical contributions further elaborated, a critical advancement is required. Previous BSMs are so far based only on the activity dimension (García-Magariño et al., 2018; Hietanen et al., 2016; Nakul et al., 2020; Nummenmaa et al., 2014; Sachs et al., 2019; Salazar-López et al., 2015; Torregrossa et al., 2018; Volynets et al., 2019), thereby completely neglecting the valence dimension. There is now a general agreement that both arousal/activity *and valence* are the most prominent underlying dimensions of every emotional experience (Colibazzi et al., 2010; Lang et al., 1993; Posner et al., 2005, 2009), and some authors emphasized that both these experiences depend on feedback from the whole body (Russell, 2003; Wiens, 2005). Thus, the previous activity-based BSM tool only covers a limited spectrum of bodily sensations. This limitation becomes evident for example when comparing the BSM for anger and pride: These two BSMs look quite similar (both show a selective activation in the upper body, see Fig. 1 in Nummenmaa et al., 2014, or Fig. 3 in this study), although there is a fundamental difference in the quality of the feeling associated with these emotions (e.g., Barrett & Bar, 2009). This example highlights that a sensation of activation is unspecific in terms of valence (it can be a pleasant or an unpleasant activation). Following the circumplex model of affect, we therefore argue that only the combination of activity and valence-related BSMs allows for a comprehensive assessment of emotions. In fact, the degree to which an experience feels pleasant or unpleasant has a strong influence on our emotional well-being and motivation. For example, experiencing loss or disappointment can be devastating, whereas experiencing success or pleasure makes us feel good and motivates us to (re)approach the rewarding situation. Moreover, chronic hypoactivity of the mesolimbic dopaminergic system that subserves pleasure and reward can lead to prevalence of negative affect and eventually result in depression or anxiety disorder (Posner et al., 2005). For these reasons, it is important to also consider the valence-related dimension in BMSs, particularly when using BSMs as “bodily sensation markers” for emotional disorders (Nummenmaa et al., 2014). The aim of the current study was therefore to (1) explore whether bodily sensations of valence are also part of emotion knowledge, so that emotion-specific BSMs of valence can be found (similar to the already established activity BSMs), and (2) to assess the added value of including both valence and activity-related BSMs in emotion research. This study therefore provides an important step in advancing the theoretical and practical value of bodily sensation mapping.

## Sensations of bodily weight as proxy for valence

A particular challenge for reaching this aim is to find an appropriate way to capture subjective valence-related sensation for specific body regions. While it is straightforward to ask for a global pleasantness–unpleasantness or positive–negative rating as in the traditional dimensional assessments of valence (e.g., on a Likert scale), we doubt that these terms describe best what people actually experience in different body parts. To illustrate, expressions such as “my chest feels negative/unpleasant” or “my head feels positive/pleasant” sound odd.^1^ Following the idea that the “wisdom” of language can help to discover the representational structure of a psychological phenomenon (e.g., Davitz, 2016; Kövecses, 2003; Oatley & Johnson-laird, 1987; Shaver et al., 1987), we considered how people describe their positive and negative feelings in language. Emotional well-being—or lack thereof—ranging from happiness on the positive side to sadness and depression on the negative side of the valence spectrum is often expressed by means of the metaphor of weight (e.g., Hamdi, 2016; Hung et al., 2017; Yu, 1995). Someone who is happy feels light-hearted, whereas someone who is sad feels *heavy*-hearted or feels *heavy* in his/her chest. Similar weight-related expressions of emotions can be found across different languages and cultures (Moradi & Mashak, 2013; Wenfeng, 2008). Do these metaphorical expressions simply reflect figurative language, or do they possibly reflect an embodied grounding of valence? Striking evidence for the latter view comes from patients suffering from depression. They often describe their emotional state with a feeling of *bodily heaviness* (Barkow et al., 2004; Fuchs & Schlimme, 2009), and antidepressant effects as *bodily lightness* (or decrease in bodily heaviness, respectively) (Stocker et al., 2019; see also van Schalkwyk et al., 2018). Some examples of patients reporting antidepressant effects are: “I had less *heaviness* in my throat and my chest”, “The *weight* was gone”, “I felt happy and *light*”, “All the pressure in my head just starts to feel *light* and normal again” (Stocker et al., 2019). If pleasant feelings of lightness and unpleasant feelings of heaviness can be attributed to specific body parts by patients suffering from depression, then maybe the valence of emotions is also embodied as sensations of weight in the non-clinical population. Zhao et al. (2016) presented healthy participant a picture of a balance as priming stimulus with a heavy and a light stone on the left and right balance pans. The same illustration was then also used to present emotional words (with positive and negative emotion words instead of stones). They found that participants were faster in judging the valence of the words when weight and valence were congruent (i.e., positive-light, negative-heavy) when compared to the opposite pairing. Such an automatic coupling between weight and valence supports the view that the use of weight may not simply reflect figurative language. Specifically, Zhao et al. (2016) concluded that the metaphorical relationship between weight and valence might be derived from bodily experiences with bodily weight and psychological weight. For example, a person may feel exhausted when holding heavy things, which would reinforce a negative mood. Also, a person feels unhappy and sad when her/his psychological burden is heavy. Moreover, happiness and pleasure are associated with the release of hormones and neurotransmitters (the so-called “happy hormones”) such as serotonin, dopamine, oxytocin, noradrenaline, and endorphins (e.g., Breuning, 2016). Some of these prepare the organism to act (e.g., dopamine and noradrenaline increase blood flow of the internal organs and muscles) and decrease pain levels during physical activity (e.g., endorphins, serotonin). Such states of increased motivation and energy help to overcome physiological and psychological barriers, and weights might appear lighter in such conditions (e.g., Farrell, 1985; Tajadura-Jiménez et al., 2015). Thus, there might be an experiential basis for the link between sensations of bodily weight and valence. This argumentation is in line with conceptual metaphor theory, according to which people often refer to familiar, tangible, and concrete concepts (source domain) to understand and express abstract concepts (target domain) (Lakoff & Johnson, 1980; Lakoff & Kövecses, 1987). As stated earlier, emotions can be considered as somewhat abstract feelings that are difficult to express (Becker‐Weidman & Hughes, 2008; Saarni, 1999). The associations between bodily weight and valence may be an illustration of conceptual metaphors where the more concrete experience of bodily weight serves as source domain used to understand and describe the more abstract domain of emotional valence, and the mapping of these domains is grounded in specific bodily sensations (Damjanovic & Santiago, 2016; Johnson, 2015; Niedenthal et al., 2005a). This is also in accordance with Borghi and Binkofski (2014)’s “Words As social Tools” view, according to which both sensorimotor and linguistic experiences form the basis of abstract concepts. Given the wide and systematic use of the weight–valence association across cultures and across clinical and non-clinical populations (Moradi & Mashak, 2013; Wenfeng, 2008), we used the concept of bodily weight (lightness, heaviness) as a proxy for valence in this study. Arguably, there are also other concepts that could potentially serve this purpose, as further elaborated in the “limitations of this study” section of the discussion.

## Outline and aims of the current study

The outline of this study was as follows: We first assessed whether specific BSMs can be created based on the feeling of bodily weight (Experiment 1). To this end, participants were asked to put themselves into different emotional states as indicated by emotion words (basic emotions: anger, fear, disgust, happiness, sadness, surprise; non-basic emotions: anxiety, love, depression, contempt, pride, shame, envy),^2^ and to mark bodily regions that change with respect to the sensation of bodily lightness or heaviness. Since valence is an inherent part of emotional feelings (Posner et al., 2005), we expected that participants will consistently associate different emotional states to specific sensations of bodily lightness and heaviness, resulting in emotion-specific “weight-BSMs”. Following the metaphorical use of weight when describing subjective feelings, we expected a systematic increase in bodily lightness for positive emotions, and an increase in bodily heaviness for negative emotions. The distinctness of the different BSMs was tested by means of a machine learning classification approach (following Nummenmaa et al., 2014).

The remaining experiments (2–4) assessed the added value of including weight BSMs in emotion research. In Experiment 2, participants created both weight and activity BSMs and indicated after each drawing how difficult they found it to express their bodily sensations in terms of these different concepts (weight, activation), and they also indicated for each emotion whether they found it clearer to express their sensations in terms of bodily activity or in terms of bodily weight. This would reveal whether activity or weight is the clearer (or dominant) bodily sensation for the different emotional states. We expected that weight BSMs are particularly appropriate to capture bodily sensations induced by emotions at the extreme poles of the valence spectrum, such as sadness and depression on the one end, and happiness on the other end. Conversely, we expected that activity BSMs are particularly suitable to capture bodily sensations induced by emotions that are associated with high arousal, such as the fight and flight-related emotions anger and fear. The knowledge of which dimension (activity or a valence-related sensation such as bodily weight) is most relevant for different emotional states is an important step for a flexible and economic use of BSMs (e.g., using different dimensions for specific emotions). We further quantified the added value of the two-dimensional BSM approach (activity *and* valence) by comparing classification performance based on weight or activity BSMs alone to the classification performance based on the combined weight and activity BSMs. Based on the circumplex model of affect, we expected that emotions are better recognized from the BSM patterns when both weight and activity information are available. This hypothesis was tested using again a machine learning approach based on the data obtained in Experiment 2, and also by asking humans to classify emotions based on BSMs (Experiment 3).

In the previous experiments (1–3), participants explicitly put themselves into different emotional states as indicated by different emotion words. It is therefore possible that semantic/linguistic associations contribute to the creation of BSMs (see “Discussion” for further elaboration of this issue). To reduce semantic influence, we aimed to replicate the pattern of results obtained in Experiment 2 for sadness induced by a sad movie scene instead of induced by the explicit emotion word in a final experiment (Experiment 4). We particularly focused on sadness, because it is a common experience in the general population and one of the main criteria of major depressive disorder (e.g., Tebeka et al., 2018).

In sum, this study explores for the first time if and how valence, as one of two fundamental aspects of emotional feelings (Posner et al., 2005), is represented with specific body topography for different emotions, and clarifies the most appropriate way (activity vs. valence) for using BSMs in emotional assessment for different emotional states.

## Experiment 1: weight-bodily sensation maps

### Method

#### Participants

189 adults participated in Experiment 1 (mean age: 25.0, ranging from 18 to 61; 158 female). Participants gave their informed consent prior to the study. They indicated by self-report that they have no history of clinical or neurological problems. The study was approved by the Ethics Committee of the University of Bern**.** Participants were recruited by means of the university’s recruiting system for undergraduate psychology students of the University of Bern and Zurich, and the link to the online study was also distributed over email and social-media channels within the social networks of the authors. About 60% of participants were undergraduate students who received course credit for participation. Data collection was stopped after a predefined time limit (end of term). The number of participants was in a similar range to previous studies (Nummenmaa et al., 2014).

#### Procedure

Data were acquired online (from remote) with the emBODY-tool developed by Nummenmaa et al. (2014) provided at https://version.aalto.fi/gitlab/eglerean/embody. In this java-based computerized tool, participants were shown two blank silhouettes of a human body (one on the left, and the other on the right side of the screen). Each body silhouette was embedded in an invisible rectangle of 171 × 522 pixels. Participants were instructed to put themselves in the emotional state indicated by the emotion word that was displayed in the center of the screen (between the silhouettes), and to focus on any sensation that they feel in their body. They were asked to paint with the mouse cursor the body regions in the body silhouette for which they felt a change in the feeling of bodily lightness/heaviness. Specifically, they painted the regions that felt heavier in the left silhouette, and the regions that felt lighter in the right silhouette. They were told that they can paint in all body regions, from head to toe. The painting was dynamic, so that successive strokes on a region increased the opacity of the paint, and the diameter of the stroke was 12 pixels. Painting in the left body silhouette was colored in purple (indicating heaviness), and painting in the right body was colored in yellow (indicating lightness). In each trial, the participants could paint as long as they wanted, and they also had the option to erase the entire painting and restart. The next trial started when participants clicked on a button at the right bottom of the screen. Six basic emotions (anger, fear, disgust, happiness, sadness, surprise),^2^ seven non-basic emotions (anxiety, love, depression, contempt, pride, shame, envy), and a neutral state (i.e., the word “neutral”) were presented in random order. The experiment lasted approximately 10 min.

#### Data analysis

Participants leaving more than mean + 3 *SD*s of bodies untouched were excluded from analysis. This was true for 4 participants. In the remaining sample (*n* = 185), participants left on average 1.3 bodies empty (this was in most cases the neutral emotion). For the construction of BSMs and analysis, we followed the procedure of Nummenmaa et al. (2014) who provided an MATLAB script at https://version.aalto.fi/gitlab/eglerean/embody. For each participant, heaviness and lightness maps for each emotion were combined into single BSMs by subtracting the values of the right silhouette (heaviness) from the values of the left silhouette (lightness). Paintings outside the body silhouette were removed. In random effects analyses, mass univariate *t* tests were then used on the subjectwise BSMs to compare pixelwise values of the BSMs for each emotional state against zero. This resulted in statistical *t *maps where pixel intensities reflect statistically significant changes in experienced feeling of bodily weight associated with each emotional state. False discovery rate (FDR) correction with an alpha level of 0.05 was applied to the statistical maps to control for false positives due to multiple comparisons.

To test whether different emotions are associated with statistically different BSMs, we used a machine learning approach (linear discriminant analysis; LDA) using the “caret”-package in R (Kuhn, 2008). Following the procedure of Nummenmaa et al. (2014), we reduced the dimensionality of the dataset to 30 principal components with principal component analysis (after first removing pixels with zero or near-zero variance). The classifier was tested using stratified 10-fold cross-validation. Accordingly, the data are shuffled and split into ten folds, trained on nine folds and tested on the remaining fold. Classification performance was assessed by means of proportion tests against chance level (either 1/7 = 14.29% for the six basic emotions and the neutral state, or 1/14 = 7.14% for the full dataset including all 13 emotions and the neutral state). The validation procedure is typically repeated several times to increase the precision of the estimates (Brownlee, 2021). We repeated the validation procedure from 1 to 10 times and observed that the estimated performance values stabilized with three repetitions. We therefore repeated the 10-fold cross-validation procedure three times for estimating the mean accuracy, standard deviation (SD), and also Cohen’s Kappa of the classifier. The latter normalizes accuracy at the baseline of random chance.

Following Nummenmaa et al. (2014), the classifier was trained separately (1) for the classification of the six basic emotions (and the neutral state) and (2) for the classification of all 13 emotions (and the neutral state) (see Footnote 2 about the definition of basic emotions).

### Results

Data of Experiment 1 are available at https://osf.io/srvhp/

**Fig. 1:**
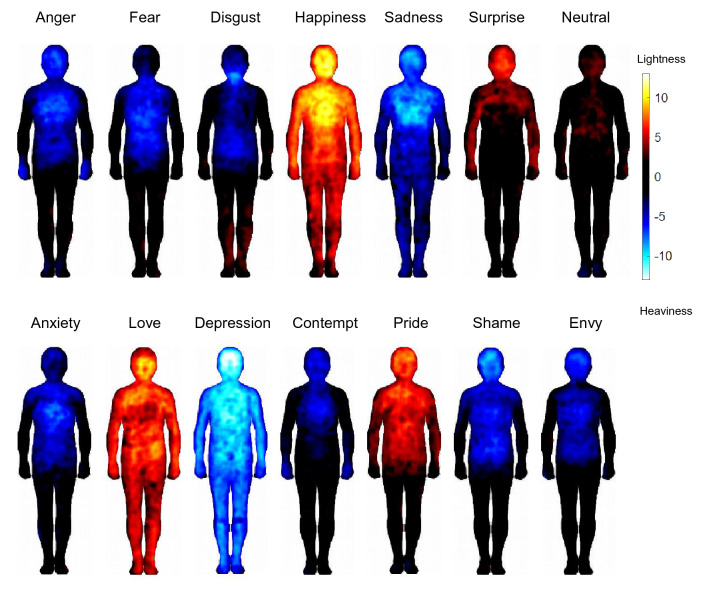
Bodily sensation maps (BSMs) of basic emotions including a neutral state (upper panel) and non-basic emotions (lower panel). The warm colors show body regions with an increased sensation of bodily lightness, and the cold colors show regions with an increased sensation of bodily heaviness when participants displaced themselves into the different emotional states. The color bar indicates the *t*-statistic range (*p* < .05, FDR correction)

The weight BSMs are shown in Fig. 1 (presented in the same arrangement as the activity BSMs in Nummenmaa et al., (2014), p. 647, allowing for convenient comparison). For each emotion, statistically significant body regions that increased in bodily lightness (warm colors) or bodily heaviness (cold colors) could be detected (*p* < 0.05; FDR corrected). Thus, participants did not draw randomly, but rather consistently marked specific body regions that they associated with feelings of lightness/heaviness in an emotion-specific way. The most salient sensation of bodily lightness was found for the most positive emotion happiness, followed by love and pride. Conversely, the most salient sensation of bodily heaviness was found for the most negatively valenced emotions depression and sadness. Specifically, there was a pronounced sensation of bodily weight in the upper body half (with a peak in the chest/heart region) for sadness, and an even more amplified and widespread sensation of bodily weight for depression, with an additional peak in the head region. Moreover, less-pronounced heaviness patterns were found for the other negatively valenced emotions anger, fear, anxiety, shame, and envy. Thus, the magnitude of lightness or heaviness in the different BSMs corresponded to the valence that is typically associated with these emotions (Posner et al., 2005, 2009; Russel, 1980), confirming that a sensation of bodily weight (lightness, heaviness) is a valid indicator of valence. Specifically, the topographic weight-BSM patterns roughly corresponded to the metaphorical use of weight when describing pleasant and unpleasant emotional feelings (see main discussion section for further interpretation of the BSM patterns).

LDA classified each of the basic emotions and the neutral state with an estimated accuracy of 43.9% (*SD* = 4.8%; Kappa = 34.5%), which was significantly better than expected by chance (chance level = 14.29%), χ^2^ = 891.07, *p* < 0.001. The highest accuracy was found for happiness (55.5%), followed by disgust (46.3%), anger (37.7%), sadness (38.0%), surprise (34.4%), and fear (23.5%).

**Fig. 2:**
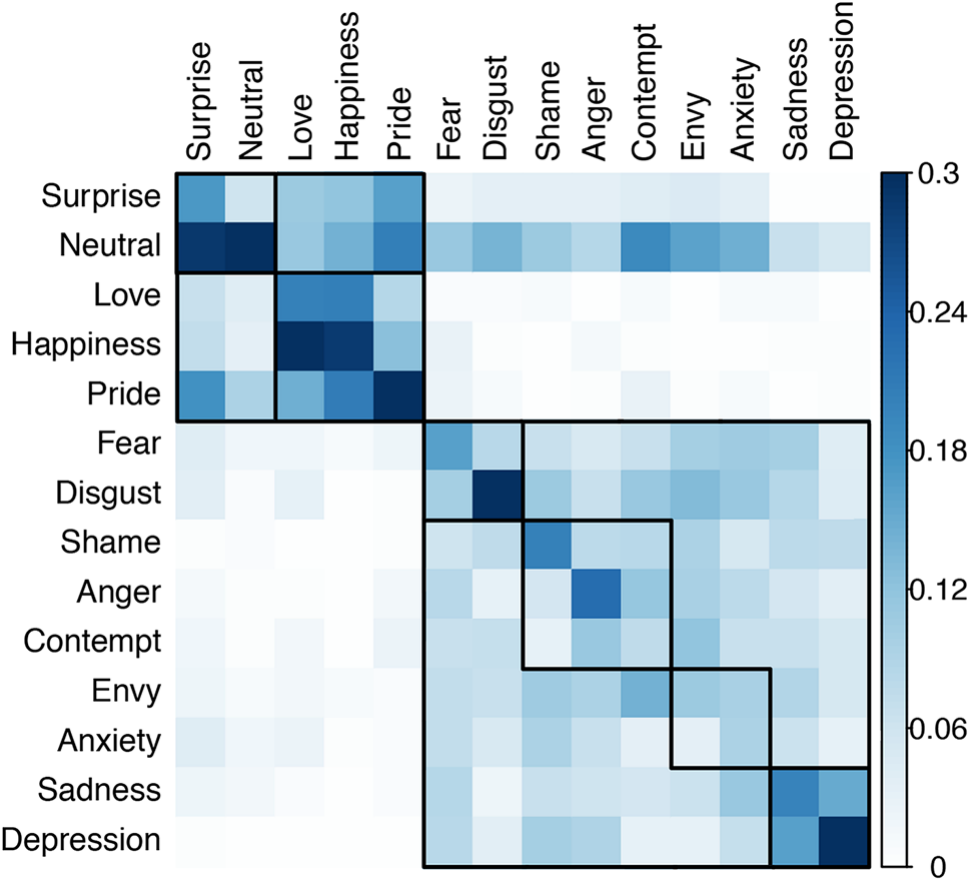
Classification matrix for the classification of all emotions (including the neutral state) against each other (chance = 0.07), ordered following hierarchical clustering (complete linkage method). Emotions along the horizontal axis reflect the true, and those along the vertical axis reflect the classified emotions (the values in each column sum up to 1). Note that dark blue means 0.3 or higher (e.g., depression with the highest hit rate of all emotions is 0.45)

The estimated classification accuracy for the full dataset (all 13 emotions and the neutral state) was 25.3% (*SD* = 2.6%, Kappa = 19.6%) and was also significantly higher than expected by chance (chance level = 7.14%), χ^2^ = 282.41, *p* < 0.001. The highest emotion-specific accuracy was found for depression (45.0%), followed by disgust (34.2%), pride (30.3%), happiness (29.2%), anger (22.7%), love (22.3%), shame (20.7%), sadness and surprise (both 18.7%), fear (16.9%), envy (11.5%), anxiety (8.6%), and contempt (8.3%). Classification matrix for the full dataset is shown in Fig. 2.

We also computed a hierarchical cluster analysis based on the classification results of the LDA. Higher and lower level clusters are marked by means of the solid squares in the classification matrix (Fig. 2). There were two high-level clusters, one with all negative emotions and one with all non-negative emotions. For the non-negative emotions, two lower level clusters were found, one including the positive emotions love, happiness, and pride, and the other including surprise and the neutral state, which are both neither positive nor negative in terms of valence. There were also lower level clusters within the negative emotions, such as fear and disgust, and sadness and depression. An example of a six-subcluster solution is shown in Fig. 2. The finding that these clusters basically reflect different categories of valence further confirms that a sensation of bodily weight (lightness, heaviness) is a valid proxy for valence.

Experiment 1 established that BSMs can be created on the basis of sensations of bodily weight (lightness, heaviness), and that the BSM patterns are distinct. The overall classification performance based on LDA found in Experiment 1 (basic emotions: 43.9%; full data set: 25.3%) was similar to the one reported in Nummenmaa et al. (2014) for activity BSMs (basic emotions: 38.0%; full data set: 24%). This suggests a similar degree of distinctness between sensations of bodily weight and activity for the different emotional states.

Since participants from Experiment 1 created only weight BSMs, we cannot yet conclude that sensations of bodily weight reflect something subjectively different than sensations of activity. It also remains unclear whether the sensations induced by the different emotions are more clearly perceived in terms of weight or activity. These questions are further addressed in Experiment 2.

## Experiment 2: weight BSMs versus activity BSMs

The aim of Experiment 2 was to compare weight and activity BSMs and particularly to assess a possible benefit of using weight BSMs. To this end, the same individuals created both weight and activity BSMs, and classification performance of LDA was compared for either weight or activity BSMs alone, and also for the combined weight and activity BSMs. Importantly, subjective ratings were collected to assess the appropriateness of both concepts (weight, activity) for each emotion. In a first step, participants rated after each BSM how easy/difficult they found it to create the BSMs. This rating will not only reveal how easy/difficult it is to draw BSMs for the different emotions, but it will also provide some hints about which concept (weight vs. activity) best captures each emotion. However, a difficulty rating alone might not be sufficient to capture more fine-grained differences in the appropriateness of the two concepts. For example, it can be equally easy to map sensations induced by pride as bodily lightness and activation on the chest and head, because the location and type of sensations (i.e., lightness in case of weight BSMs, and activation in case of activity BSMs) is easy to identify in both cases. Despite equal difficulty, it is still possible that one of the concepts better represents what a person actually feels (weight or activity). We believe that differences between the two concepts become most apparent when participants are forced to explicitly compare the two concepts against each other. In a second step, we therefore also asked participants to indicate the clarity with which they experience and express their bodily sensations by both concepts on a scale with lightness/heaviness on one end and activation/deactivation at the other end. Apparently, such a direct comparison is only possible after participants have displaced themselves into the different emotional states and completed the weight and activity BSMs. Therefore, the clarity rating was asked only after all BSMs have been completed.

In contrast to Experiment 1 where data were collected online, all data for Experiment 2 were collected in a laboratory setting.

### Method

#### Participants

Sixty-seven new adults participated in this study (mean age: 24.6, ranging from 18 to 55; 44 female). Since one of the main aims of Experiment 2 was to find differences in clarity ratings between a feeling of weight and activity (which is assessed by a one-sample *t* test against 50), the sample size for Experiment 2 was based on a two-tailed one-sample *t* test with a small-to-medium-effect size (*d* = 0.35), a power of 0.8, and an alpha level of 0.05 using g-power (Faul et al., 2007). Participants gave their informed consent prior to the study. The study was approved by the Ethics Committee of the University of Bern. Participants were recruited by means of the participants’ pool for undergraduate psychology students of the University of Bern, and a research assistant additionally recruited participants from her circle of acquaintances. About 80% of participants were undergraduate students who received course credit for participation. All participants reported by self-report that they have no history of clinical or neurological problems.

#### Procedure

In contrast to Experiment 1, in Experiment 2, participants were presented with each emotion word twice, once with the same instruction as described in Experiment 1 (lightness/heaviness), and once with the original instruction used in Nummenmaa et al. (2014). Specifically, for activity BSMs, participants were instructed to indicate the body regions in the left silhouette that felt more activated (i.e., stronger, faster) than usual, and in the right silhouette body regions that felt more deactivated (weaker, slower) than usual. The 28 trials (14 different emotions × 2 instructions) were presented in random order. After the completion of each BSM, participants indicated on an analog scale how difficult they found it to mark their sensations on the body map (scale ranging from very easy to very hard). Moreover, after all 28 BSM trials were completed, participants were asked to put themselves again into each emotional state and to indicate whether they find it clearer to experience/describe their sensation as activation/deactivation or as a feeling of lightness/heaviness. To this end, the 14 emotion words were again presented in random order along with an analog scale with the left end marked as “clearer with activation/deactivation” and the right end marked as “clearer with lightness/heaviness”.

To implement the modifications used for Experiment 2 (two different instructions (weight, activity), difficulty ratings following each BSM, clarity rating at the end of the BSMs), we used a different application for Experiment 2 (Experiment Builder, SR-Research), whereby the appearance of the BSM trials was very similar to the original emBODY-tool used in Experiment 1. Stimuli were presented on a 15-inch laptop with a resolution of 1024 × 768 pixels. The body silhouettes had a size of 171 × 522 pixels and were positioned + /– 367 pixels from the center of the screen. The emotion word was presented at the center of the screen with a size of 40 pixels, and the instructions (lightness/heaviness vs. activation/deactivation) appeared below the emotion word with a size of 14 pixels. The head-to-screen distance was approximately 60 cm. Each session lasted approximately 25 min.

#### Data analysis

BSMs were created in the same way as in Experiment 1. The difficulty ratings ranged from 0 to 100 (0 = very easy; 100 = very difficult). Difficulty ratings were analyzed by means of an analysis of variances (ANOVA) with the variables concept (weight, activity) and emotion (all 13 emotions and the neutral state), followed by paired *t* tests for each emotion (weight vs. activity). The clarity ratings also ranged from 0 to 100 (0 = clearer with activity, 100 = clearer with weight). The clarity ratings were also analyzed first by means of a repeated measure ANOVA with the variable emotion (all 13 emotions and the neutral state), followed by the critical one-sample *t* tests against 50 for each emotion (50 indicates no difference in clarity between weight and activity). *P* values of the *t* tests were adjusted for multiple comparison following Bonferroni–Holm.

Classification performance of the LDA was assessed in the same way as in Experiment 1, separately for the weight and activity BSMs. In addition to the separate analyses, we also analyzed the classification performance when both weight and activity patterns were jointly used for classification, and compared the combined classification performance to the separate weight and activity classification performance. Because the separate LDA classification of the weight and activity BSMs was based on the first 30 components following principal component analysis (see Experiment 1), the combined classification training was also based on 30 components (to allow for a fair comparison), with the 15 first principal components of the weight BSMs and the 15 first principal components of the activity BSMs. As in Experiment 1, statistical tests were based on proportion tests (one-sample proportion test against chance and two-sample proportion tests for the pairwise comparisons).

### Results

Data and supplemental material of Experiment 2 are available at https://osf.io/srvhp/

**Fig. 3:**
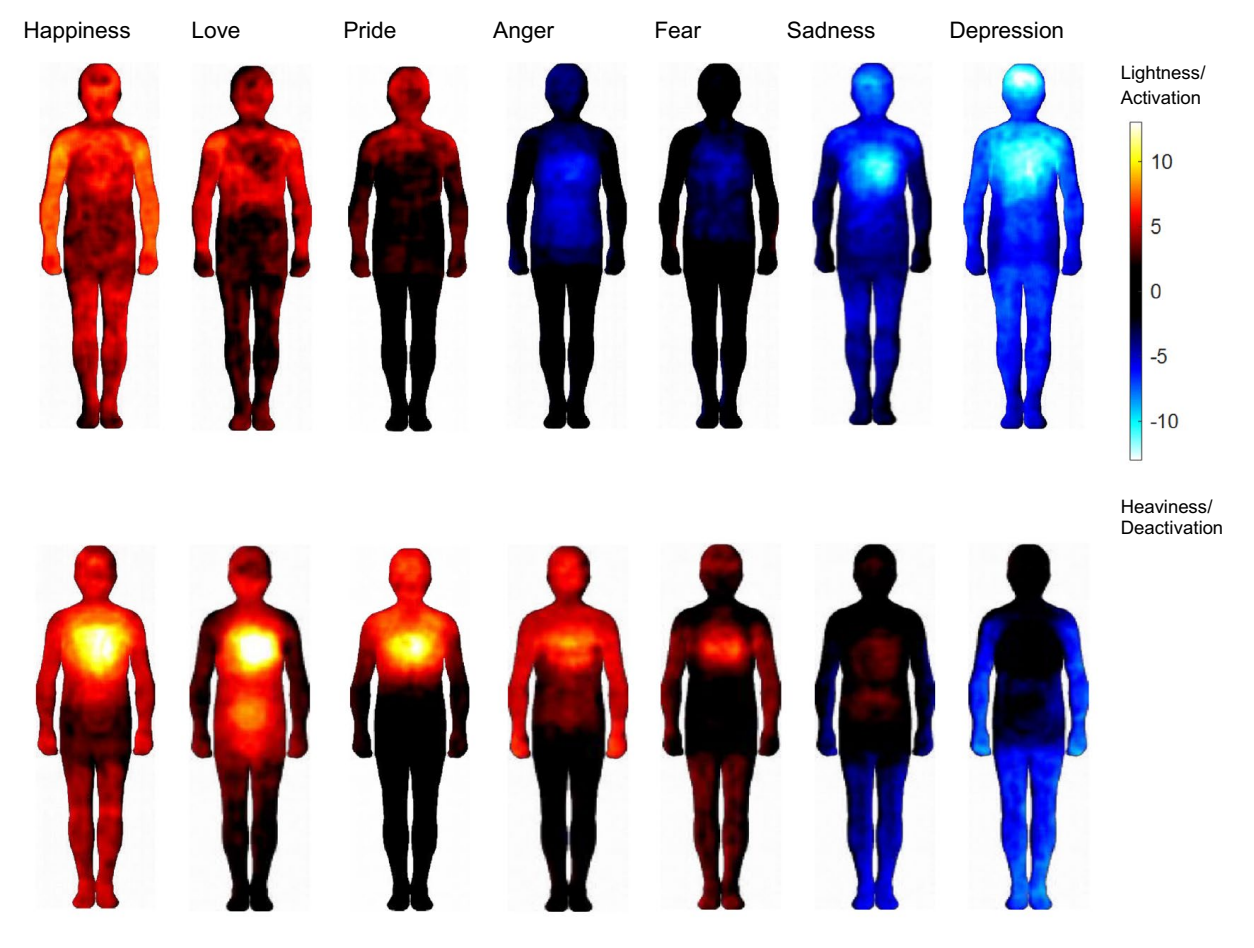
Weight (upper panel) and activity (lower panel) BSMs for some representative emotions. For weight BSMs, warm colors indicate a feeling of lightness, and cold colors a feeling of heaviness. For activity BSMs, warm colors indicate bodily activation, and cold colors bodily deactivation. The colors at the endpoints of the color bar indicate that at least 36 (out of 67) participants reported a change in the feeling of lightness or activation (yellow) or in the feeling of heaviness or deactivation (light blue) for the respective body parts

No participant was excluded due to omissions. BSMs for a selection of relevant emotions are presented in Fig. 3 (see supplemental material Figure S1 for all 2 × 14 BSMs). The weight BSMs from Experiment 2 appear very similar to the weight BSMs of Experiment 1. Moreover, the activity BSMs of Experiment 2 look similar to the activity BSMs reported in Nummenmaa et al. (2014). This suggests that the weight and activity BSMs reflect stable and reproducible patterns within and across studies. For the positive emotions (happiness, love), the bodily sensations are experienced as *activation and lightness*. In contrast, the bodily sensations associated with anger, fear, and sadness (for the head/chest/belly region) are experienced as *activation and heaviness*, while the bodily sensations associated with depression are experienced as *deactivation and heaviness*. Thus, depending on the valence of the emotional feeling, bodily sensations of activation can be experienced at the same time as lightness or heaviness, highlighting that only activation and valence in combination provide a comprehensive picture of BSMs.

#### Difficulty ratings

The overall mean difficulty rating was 44.7 (*SEM* = 2.6), and 10 out of the 14 emotion-specific means were below 50, suggesting that participants found it in general rather easy than difficult to create BSMs. The ANOVA revealed no main effect of concept, *F*(1,66) = 1.35, *p* = 0.249, η^2^_p_ = 0.02, indicating that it was overall equally difficult (or easy) to create weight BSMs (45.4, *SEM* = 1.5) and activity BSMs (*M* = 44.2, *SEM* = 1.6). The main effect of emotion indicated that it was not equally difficult (or easy) to create BSMs for the different emotions, *F*(13,858) = 21.50, *p* < 0.001, η^2^_p_ = 0.25, and there was also an interaction between concept and emotion, *F*(13,858) = 3.25, *p* < 0.001, η^2^_p_ = 0.05. The mean difficulty rating for each emotion is shown in Table 2 (ordered according to their mean difficulty rating), with happiness and love as the easiest emotions, and envy and contempt as the most difficult emotions for creating BSMs. The comparison between the two concepts (weight, activity) for each emotion revealed that, after Bonferroni–Holm adjustment, there was only a significant difference for disgust (see Table 1).

**Table 1:** Difficulty ratings for the weight and activity BSMs

Emotion	Overall	Weight	Activity				95% CI		
	*M* (SEM)	*M* (SEM)	*M* (SEM)	*M*_Diff_	*t*	*p*	Lower	Upper	Cohen's d
Happiness	28.9 (2.3)	26.9 (2.5)	31.0 (2.9)	4.1	1.58	1.000	− 1.09	9.3	0.19
Love	30.0 (2.4)	31.2 (2.8)	28.7 (2.6)	− 2.5	− 0.94	1.000	− 7.76	2.81	− 0.11
Anger	38.4 (2.4)	39.9 (2.8)	37.0 (2.9)	− 2.9	− 0.98	1.000	− 8.74	3.01	− 0.11
Depression	39.6 (2.8)	36.7 (3.2)	42.6 (3.1)	5.9	1.99	0.606	− 0.01	11.86	0.24
Sadness	40.3 (2.1)	39.3 (2.7)	41.3 (2.7)	2.0	0.58	1.000	− 4.83	8.77	0.07
Pride	40.4 (2.6)	41.0 (2.9)	39.9 (2.9)	− 1.0	− 0.39	0.699	− 6.41	4.32	− 0.04
Fear	42.9 (2.3)	45.8 (2.7)	40.1 (2.8)	− 5.7	− 1.87	0.726	− 11.7	0.4	− 0.23
Anxiety	46.4 (2.2)	49.9 (2.8)	42.8 (2.5)	− 7.2	− 2.37	0.273	− 13.17	− 1.13	− 0.29
Surprise	48.2 (2.4)	50.1 (2.7)	46.3 (2.8)	− 3.8	− 1.31	1.000	− 9.57	1.99	− 0.16
Disgust	48.9 (2.4)	55.4 (2.9)	42.4 (2.8)	− 13.0	− 4.27	< .001	− 19.1	− 6.93	− 0.52
Shame	50.2 (2.6)	51.0 (2.9)	49.3 (3.1)	− 1.7	− 0.53	1.000	− 8.16	4.73	− 0.06
Neutral	51.0 (3.3)	49.9 (3.7)	52.1 (3.7)	2.2	0.66	1.000	− 4.36	8.69	0.08
Envy	58.6 (2.5)	57.8 (2.9)	59.3 (2.8)	1.5	0.53	1.000	− 4.21	7.25	0.06
Contempt	62.9 (2.3)	60.5 (2.8)	65.4 (2.6)	4.9	1.72	0.910	− 0.81	10.63	0.21

Emotions are ordered following the mean difficulty ratings (low values = easy, high values = difficult). *P* values are adjusted for multiple comparisons using the Bonferroni–Holm method

#### Clarity ratings

**Fig. 4:**
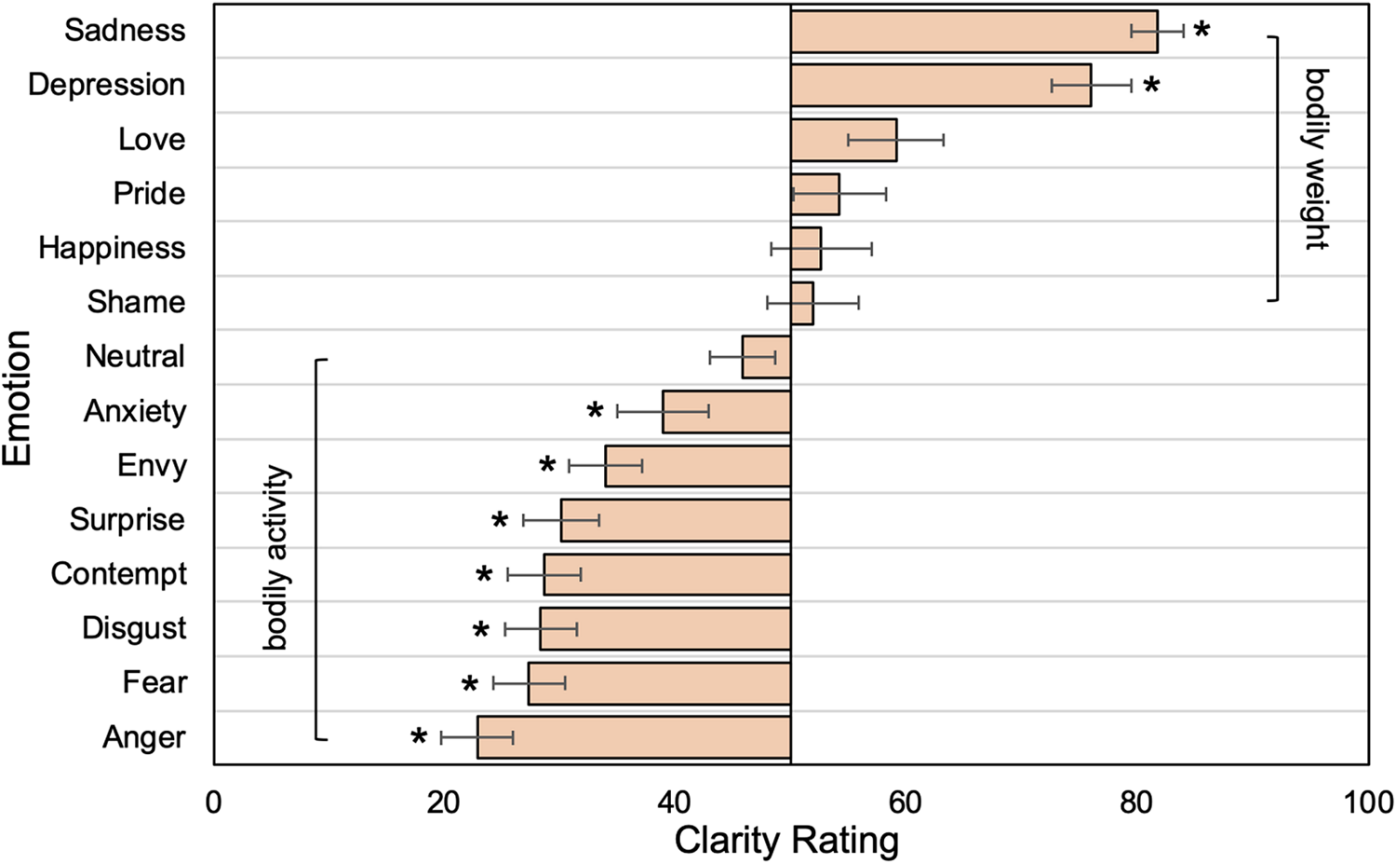
Mean clarity ratings for each emotion. The vertical line (clarity = 50) means no difference in clarity between the two bodily sensations (weight vs. activity). Asterisk indicate significant preferences (*p* < .05 following Bonferroni–Holm adjustment) for perceiving and expressing emotions in terms of bodily weight (right side) or in terms of bodily activity (left side). Error bars depict + /– 1 *SEM*

The ANOVA revealed that the clarity to experience and express an emotion in terms of bodily weight or bodily activity differs across emotions, *F*(13,858) = 29.50, *p* < 0.001, η^2^_p_ = 0.31. The comparison of the clarity ratings of each emotion against 50 (no difference in clarity between weight and activity) revealed that the sensations induced by sadness and depression were clearer experienced and expressed in terms of bodily weight than in terms of bodily activity (sadness: *M* = 81.7, *SEM* = 2.3; *t*(66) = 14.11, *p* < 0.001, Cohen’s *d* = 1.72, 95% CI [27.2, 36.2]; depression: *M* = 76.0, *SEM* = 3.4; *t*(66) = 7.60, *p* < 0.001, Cohen’s *d* = 0.93, 95% CI [19.2, 32.8]). The opposite was true for the emotions anxiety *(M* = 38.9, *SEM* = 4.0; *t*(66) = − 2.79, *p* = 0.041, Cohen’s *d* = − 0.34, 95% CI [− 19.1, − 3.1]), envy *(M* = 33.9, *SEM* = 3.2; *t*(66) =  − 5.03, *p* < 0.001, Cohen’s *d* = − 0.61, 95% CI [− 22.5, − 9.7]), surprise *(M* = 30.1, *SEM* = 3.3; *t*(66) =  − 6.09, *p* < 0.001, Cohen’s *d* = − 0.74, 95% CI [− 26.4, − 13.4]), contempt (*M* = 28.6, *SEM* = 3.2; *t*(66) =  − 6.76, *p* < 0.001, Cohen’s *d* = − 0.83, 95% CI [− 27.7, − 15.1]), disgust (*M* = 28.3, *SEM* = 3.1; *t*(66) =  − 6.97, *p* < 0.001, Cohen’s *d* = − 0.85, 95% CI [− 28.0, − 15.5]), fear (*M* = 27.3, *SEM* = 3.2; *t*(66) =  − 7.21, *p* < 0.001, Cohen’s *d* = − 0.88, 95% CI [− 29.0, − 16.5]), and anger (*M* = 22.8, *SEM* = 3.1; *t*(66) =  − 8.69, *p* < 0.001, Cohen’s *d* = − 1.06, 95% CI [− 33.4, − 20.9]; see Fig. 4). No preference was found for love, pride, happiness, shame, and the neutral state (all *p*s > 0.05 following Bonferroni–Holm correction).

#### LDA performance

**Fig. 5:**
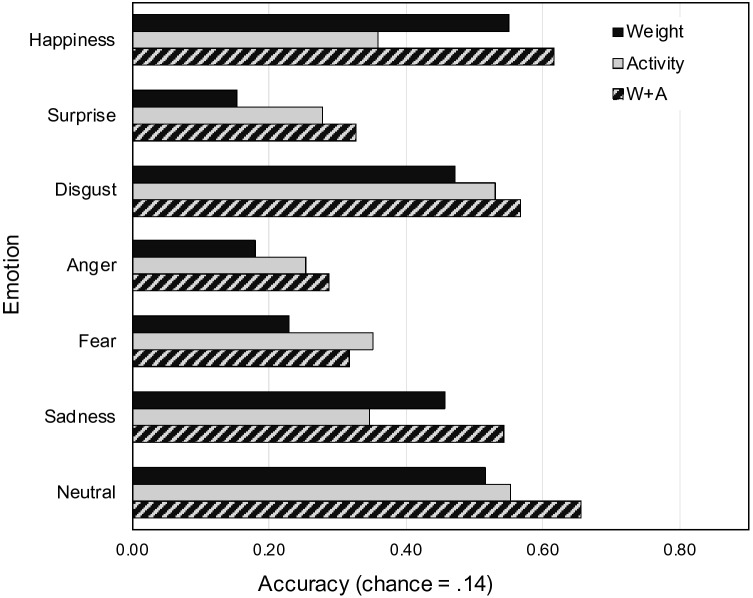
Classification performance of the LDA for the six basic emotions and the neutral state. Black bars show accuracy for weight BSMs, grey bars for activity BSMs, and the black–grey bars show accuracy when classification is based on both weight and activity BSMs

LDA classification performance was very similar for the weight and activity BSMs, and was significantly higher than expected by chance for both types of BSMs (activity and weight), as summarized in Table 2. Importantly, when using both weight and activity information for emotion classification, the accuracy significantly increased when compared to the classification accuracy of weight or activity BSMs alone, see Table 2. For example, the accuracy for correctly classifying the six basic emotions and the neutral state increased roughly from ≈ 37% to ≈ 47%, corresponding to a proportional increase of ≈ 27% in performance. This confirms that assessing both activity and weight sensations significantly increases the distinctness of bodily sensations for different emotional states. The classification matrices for all conditions can be found in the supplemental material (Figure S2 and S3). The classification accuracy for the six basic emotions and the neutral state for the three different classifiers (weight vs. activity vs. activity + weight) is shown in Fig. 5 for a direct comparison. In line with our hypothesis, the classification based on the weight BSMs outperforms the classification based on the activity BSMs for happiness and sadness, and the combined classification showed the best performance for these emotions. This descriptive tendency was true for all six basic emotions except fear.

**Table 2:** Classification accuracy of the linear discriminant analysis (LDA)

BSM	Six basic emotions and neutral state (Chance = 14.3%)				All 13 emotions and neutral state (Chance = 7.1%)			
	Accuracy (SD)	Kappa	χ^2^	*p*	Accuracy (SD)	Kappa	χ^2^	*p*
Weight (W)	36.5% (7.2)	25.9%	210.57	< .001	22.9% (4.2)	17.0%	330.07	< .001
Activity (A)	38.2% (7.1)	27.9%	238.22	< .001	23.5% (4.8)	17.6%	368.06	< .001
W + A	47.4% (8.7)	38.7%	393.73	< .001	28.3% (5.5)	22.7%	626.65	< .001
W vs. A	–	–	0.22	.639	–	–	0.19	.660
W vs. W + A	–	–	8.45	.004	–	–	8.23	.004
A vs. W + A	–	–	5.95	.015	–	–	5.91	.015

Statistical values (χ^2^, *p*) are based on one-sample proportion test against chance in the upper half of the table, and on two-sample proportion tests for the pairwise comparisons in the lower half of the table

To further assess the benefit of using valence-related BSMs in emotion research, we assessed how well humans are able to recognize emotions based on the BSM of others depending on the different information regarding bodily sensations (weight vs. activity vs. weight + activity).

## Experiment 3: Human classification accuracy

### Method

#### Participants

One hundred and ten undergraduate psychology students participated in this online study (mean age: 22.8, ranging from 18 to 40; 86 female). They were recruited by means of the university’s recruitment system. None of the participants took part in the previous experiments. All participants reported by self-report that they have no history of clinical or neurological problems. Upon registration, participants received an invitation link for the experiment and they could start whenever they wanted. They received course credit for participation. Participants gave their informed consent prior to the study. The study was approved by the Ethics Committee of the University of Bern.

#### Material and procedure

We used the statistical BSMs obtained in Experiment 2 for the classification task (see supplemental material Figure S4 for the stimulus material; https://osf.io/srvhp/). For Experiment 3, we excluded the emotions envy and contempt. The reason is that participants indicated in Experiment 2 that they found it rather difficult to indicate their bodily sensations for these two emotions (see Table 1), and also the statistical BSMs (Figure S1) for these emotions showed that the bodily sensations associated with these two emotions are rather subtle. Note also that Nummenmaa et al. (2014) only included the basic emotions for their human classification experiment with the activity BSMs.

Participants were informed that they will see BSMs associated with different emotions, and that their task was to allocate the different BSMs to the corresponding emotions. They were informed that warm colors represent bodily activation (or a sensation of bodily lightness), and cold colors bodily deactivation (or a sensation of bodily heaviness) for the activity BSMs (or, respectively, for the weight BSMs).

Participants performed two emotion classification tasks. For half of participants, the weight BSMs were presented in the first task, and the combined (activity and weight) BSMs in the second task. For the other half of participants, the activity BSMs were presented in the first task, and the combined (activity and weight) BSMs in the second task.

The experiment was run on the open source survey tool Limesurvey (2012) (www.limesurvey.org). The appearance of the classification task is illustrated in the supplemental material (Figure S4, https://osf.io/srvhp/). The BSMs were presented along with a number (1–12) in the upper part of the web page (6 × 2 grid of 530 × 450 pixels for the first task, and 1076 × 450 pixels for the second task). In the lower part of the page (below the BSMs), a table was presented with numbered lines (1–12, numbers representing the BSMs) and emotion labels in colons (alphabetically ordered). Participants were asked to indicate for each BSM, the emotion they think matches best by ticking a box in the corresponding cell of the table. The second task was identical to the first except that the two BSMs (activity and weight) were presented for each emotion in the grid.

### Results and discussion

Data of Experiment 3 are available at https://osf.io/srvhp/

The classification performance of human classifiers was significantly higher than expected by chance in all BSM conditions (weight, activity, weight + activity) as summarized in Table 3. This further confirms that BSMs reflect distinct emotion-specific sensations that are part of the emotion knowledge. Interestingly, classification performance based on weight BSMs (39.5%) was significantly higher when compared to the classification performance based on activity BSMs (28.8%). Moreover, classification performance based on both weight and activity BSMs (45.2%) was significantly higher when compared to the classification based on either weight or activity BSMs alone (see Table 3).

**Table 3:** Human classification accuracy

BSM	11 emotions and neutral state (Chance = 8.3%)		
	Accuracy	χ^2^	*p*
Weight (W)	39.5%	837.62	< .001
Activity (A)	28.8%	358.81	< .001
W + A	45.2%	2347.30	< .001
W vs. A	–	16.98	< .001
W vs. W + A	–	5.78	.016
A vs. W + A	–	49.65	< .001

Statistical values (χ^2^, *p*) are based on one-sample proportion test against chance in the upper half of the table, and on two-sample proportion tests for the pairwise comparisons in the lower half of the table

**Fig. 6:**
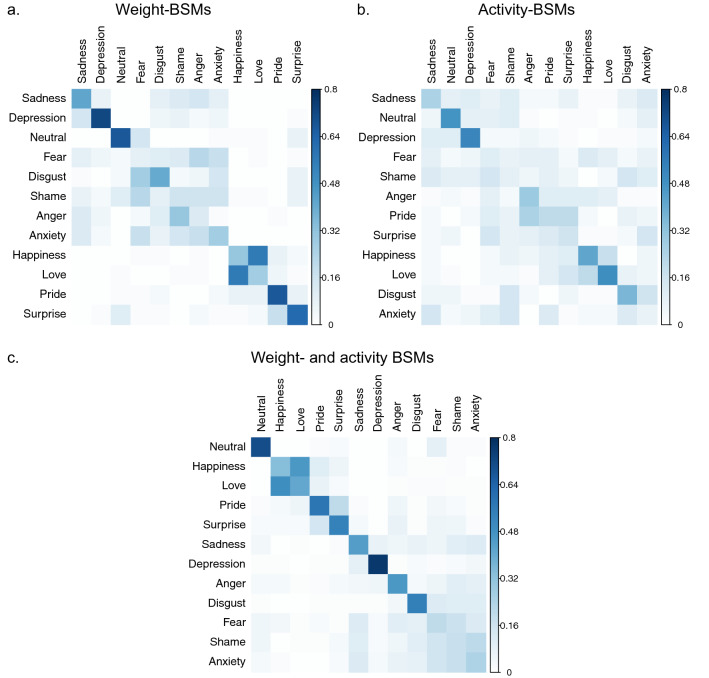
Classification matrices of humans from Experiment 3 for 11 emotions and the neural state when classification was based on weight BSMs (**a**), activity BSMs (**b**), or both BSMs (**c**). Emotions along the horizontal axis reflect the true, and those along the vertical axis reflect the classified emotions (the values in each column sum up to 1). The chance level was .083 (1/12). The order of the emotions in each figure (**a**–**c**) is determined by hierarchical clustering

**Fig. 7:**
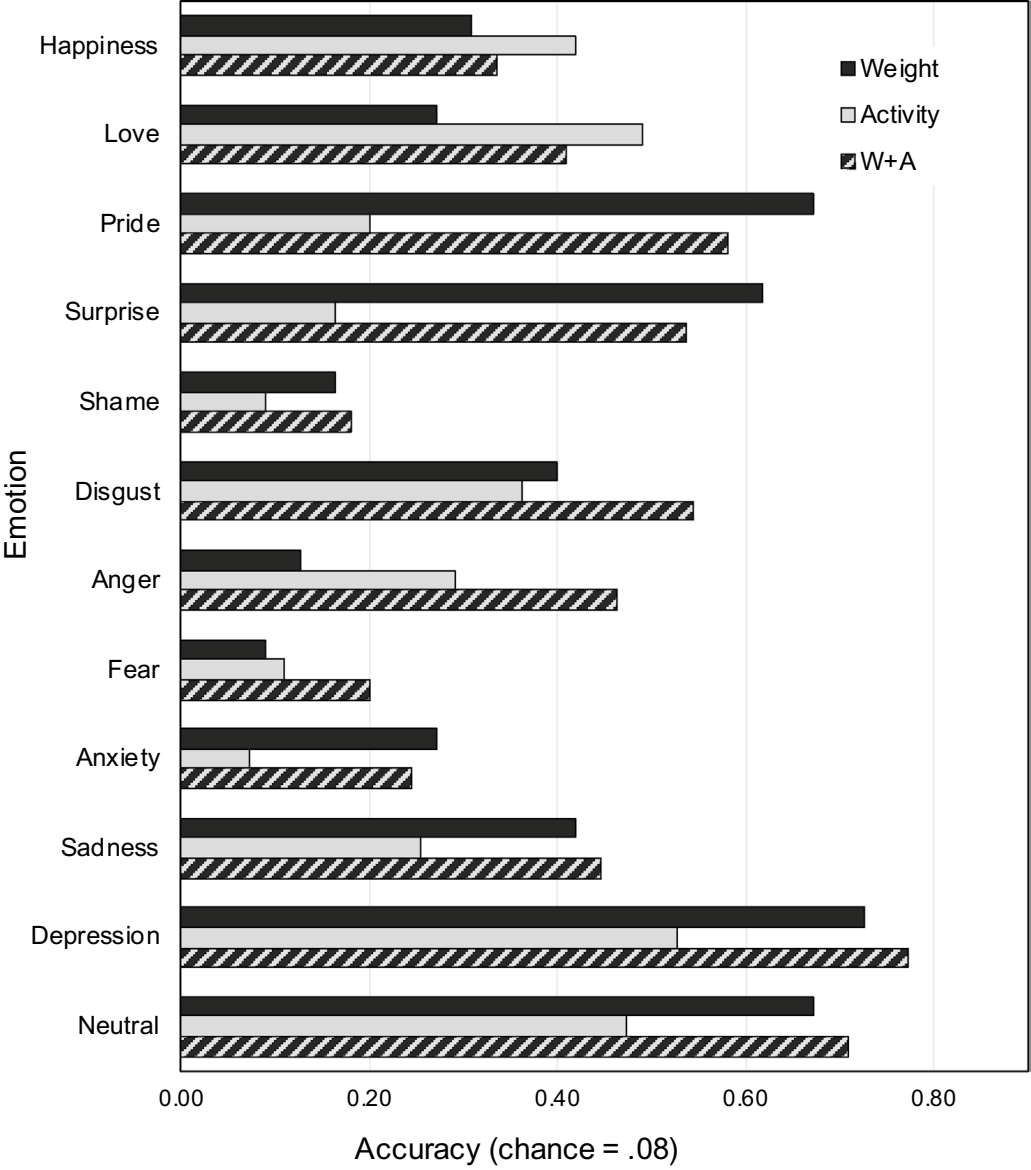
Classification performance when humans classify 11 emotions and the neutral state based on the BSMs of others. Black bars show accuracy for weight BSMs, grey bars for activity BSMs, and the black–grey bars show accuracy when classification is based on both weight and activity BSMs

Classification matrices are shown in Fig. 6. Visual inspection of the matrices reveals that the number of misclassifications (i.e., the “noise” around the diagonal line) was reduced when classification was based on both weight and activity BMSs (Fig. 6c) when compared to weight (Fig. 6a) or activity (Fig. 6b) BSMs alone. For a direct comparison, classification performance for each emotion is shown in Fig. 7. The superiority of the combined (vs. separate) classification was mainly driven by the emotions anger, fear and disgust. Thus, the combined information about sensation of bodily weight and activity makes these emotions more clearly recognizable. For example, as we have outlined in the introduction, the activity BSM for anger looks similar than the one for pride, which makes it difficult to correctly classify anger without additional valence information. For other emotions such as sadness, depression, anxiety, pride, and surprise, the sensation of bodily weight seems to be sufficient to recognize these emotions, so that the additional information about bodily activity does not further increase the classification accuracy. Conversely, for happiness and love, the sensation of bodily activity seems to be sufficient to recognize these emotions, so that the additional information about a sensation of bodily weight does not further improve the classification performance.

This pattern of result is slightly different to the one of the LDA where the classification performance was highest for the combined BSMs for most emotions. This might be related to the fact that humans, unlike computers, often rely on one salient cue (weight or activity BSM) when making decisions (Gigerenzer & Goldstein, 1999), and might also apply some strategic behavior for classification (see main discussion). It should also be noted that the classification performance for specific emotions depends on the presence (or absence) of other emotions in the set, and we used a slightly different set of emotions in this experiment.

Before we discuss the results from Experiment 2 and 3 in more detail, we aimed to replicate some of the main findings in a further experiment. Since the emotions induction in Experiment 1 and 2 were based on emotion words, it is possible that participants merely reported language-based stereotypes of emotion-specific bodily sensations rather than actual bodily sensations (Barrett, 2006; Nummenmaa et al., 2014; Rimé et al., 1990). In a final experiment, we overcome this limitation by inducing emotions without presenting corresponding emotion words. Instead, we used one of the most powerful emotion induction techniques—viewing emotional movie clips (Lench et al., 2011). For this final experiment, we focused on sadness for several reasons. First of all, most people occasionally experience sadness, since it is a common response to distressing and discouraging life events (e.g., separation, loss, failure, illness). Moreover, sadness is an integral part of depression (Mouchet-Mages & Baylé, 2008). The recognition and measurement of sadness has therefore a high relevance for therapeutic evaluation and assessment, both for the non-clinical and especially for the clinical (depressive) population. In line with our hypothesis, participants from Experiment 2 indicated that the sensations induced by sadness are clearer experienced and expressed in terms of bodily weight than in terms of activation. To further evaluate the generalizability of these findings, it is important to test whether the same results are also observed in a setting in which emotions are induced by an intense stimulus (rather than self-induced by the participant).

## Experiment 4: weight and activity BSMs induced by a sad movie scene

### Method

#### Participants

The primary aim of this study was to replicate the effect of the clarity rating for sadness. The definition of the sample size was therefore based on the two-tailed one-sample t test. According to a-priori power analysis using g-power (Faul et al., 2007), a minimum sample size of 33 is required to find medium-effect size effects (0.5) with a power of 0.8 and an alpha level of 0.05. Thirty-five undergraduate students participated in this study (mean age: 38.0, ranging from 23 to 54; 11 missing age values, 26 female). Participants gave their informed consent prior to the study. The study was approved by the Ethics Committee of the University of Bern. None of the participants took part in the previous experiments.

#### Material

A sad clip from the movie “My sister’s keeper” (Cassavetes, 2009) was chosen, which has been proven as sad and touching in previous studies (e.g., Cavanagh et al., 2015; Scrimin & Mason, 2015). The 6-min clip portrays a mother's last conversation with her dying teenage daughter, and concludes with the girl's death. The mother was portrayed by Cameron Diaz, and the daughter by Sofia Vassilieva. The clip was presented in German. Due to a different experimental setting (group assessment in a lecture room; see procedure), BSMs, difficulty, and clarity ratings were assessed by means of a paper-and-pencil version (see “Experiment 4 paper pencil version.doc” at https://osf.io/srvhp/.

#### Procedure

The experiment was conducted in a lecture room as a voluntary part of a bachelor-level introductory psychology course. Participants were informed prior to the experiment that the experiment is about emotional assessment, and that the scene is about a mother talking to her dying daughter. The movie scene was presented with an ordinary beamer on the screen in front of the room (300 × 228 cm). Depending on the student’s seating position, the distance to the screen varied between 4 and 10 m. For the sake of simplicity, we applied a paper-and-pencil version of the task. When the movie was finished, participants were asked to indicate on a paper-and-pencil version of BSMs to mark the body regions that felt different after watching the movie with respect to the feeling of bodily weight (lightness or heaviness), or with respect to the feeling of bodily activity (activation, deactivation). The layout of the body silhouette was the same as in the computerized versions used in Experiment 1 and 2: feelings of heaviness and activation were marked on the left silhouette, and feelings of lightness and deactivation were marked on the right silhouette. The two different instructions with two empty bodily silhouettes for each instruction were printed on two separate sheets of paper (one sheet for the activity-BSM, and one sheet for the weight-BSM). After they marked the bodily regions, they were asked to indicate how difficult they found it to perceived and express feelings of bodily weight, or bodily activity, respectively, on a visual analog scale that was printed below the bodily silhouette (with the left endpoint labeled as “very easy”, and the right endpoint labeled as “very difficult”). The order of the two instructions (weight or activity) was counterbalanced across participants. After they completed the two BSMs (and the associated difficulty ratings), they were asked to focus again onto their bodily sensations induced by the movie and rate the clarity with which they experienced and expressed their bodily sensations by both concepts (weight, activity). Responses were given on a visual analog scale printed on a third sheet, with the left endpoint labeled as “clearer with activation/deactivation”, and with the right endpoint labeled as “clearer with bodily lightness/heaviness”.

#### Data analysis

Difficulty and clarity ratings on the paper were quantified by a ruler, and the measurements (in mm) were converted into values from 0 to 100 by a naïve research assistant. As in Experiment 2, difficulty ratings were assessed by means of a paired sample *t *test, and clarity rating by means of a one-sample *t *test against 50 (no difference in clarity). Moreover, for descriptive reasons, the paper-and-pencil BSMs were transmitted to the computerized version by a naïve research assistant. Due to the lower sample size, we reported “descriptive” rather than “statistical” BSMs for Experiment 4. Consequently, the colors of the BSM patterns do not reflect the *t*-statistic range (as in Experiment 1 and 2) but rather the number of participants who indicated a sensation of heaviness or lightness in the respective body parts.

### Results

Data of Experiment 4 are available at https://osf.io/srvhp/

The mean clarity rating was 60.9 (*SEM* = 4.9) and differed significantly from 50, *t*(34) = 2.22, *p* = 0.033, Cohen’s *d* = 0.38, 95% CI [0.9, 20.8]. This confirms the finding from Experiment 2 that it was clearer to experience and express the bodily sensations induced by the movie (i.e., sadness) in terms of bodily weight than in terms of bodily activity. The mean difficulty rating for the weight BSM was 47.4 (*SEM* = 4.3), and 50.2 (*SEM* = 4.6) for the activity BSM. The paired *t* test revealed that this difference was not significant, *t*(33) = 0.69, *p* = 0.492, Cohen’s *d* = 0.12, 95% CI [− 5.5, 11.1].

Remarkably, the preference for bodily weight over bodily activity in the clarity ratings for sadness was less pronounced in Experiment 4 when compared to Experiment 2 (60.9 vs. 81.7, *p* < 0.001). Moreover, participants from Experiment 4 tended to indicate higher difficulty ratings than participants from Experiment 2 (activity: 50.2 vs. 41.3, *p* = 0.077; weight: 47.4 vs. 39.3, *p* = 0.100). A possible explanation for the trends toward lower difficulty ratings in Experiment 2 could be a practice effect, because participants in Experiment 2 completed several BSMs, while participants in Experiment 4 completed only two. One could also speculate that it is easier to draw bodily sensations on a computer screen than on paper. Regarding the clarity rating, it is conceivable that the preference for weight over activity is more pronounced when the emotion is induced by the emotion word than by a movie, because words might increase the role of language-based semantic associations between weight and sadness (cf. Discussion).

**Fig. 8:**
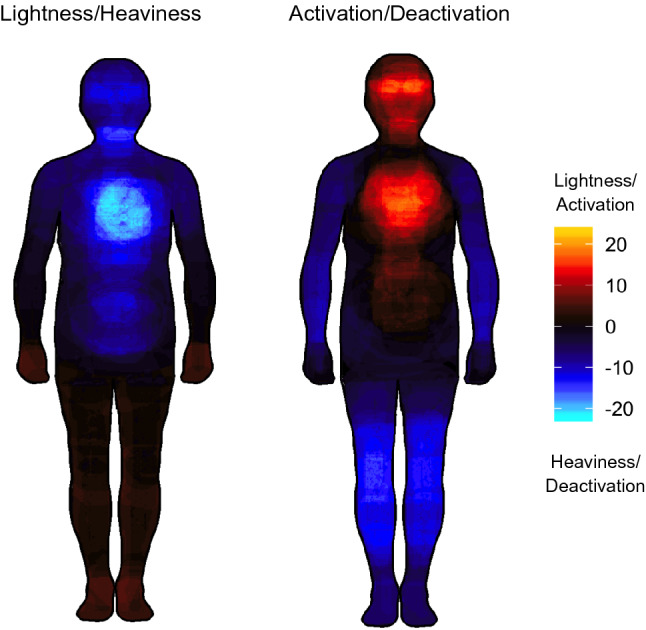
Weight (left panel) and activity (right panel) BSMs for the sensations induced by a sad movie. For weight BSMs, warm colors indicate a feeling of lightness, and cold colors a feeling of heaviness. For activity BSMs, warm colors indicate bodily activation, and cold colors indicate bodily deactivation. The colors at the endpoints of the color bar indicate that at least 23 (out of 35) participants reported a change in the feeling of lightness or activation (yellow) or in the feeling of heaviness or deactivation (light blue) for the respective body parts

Descriptive BSMs are presented in Fig. 8. The pattern of the BSMs highly resembles that of sadness from Experiment 1 and 2 (deactivation and heaviness in the chest regions).

### Discussion

The aim of this series of experiments was to advance the theoretical and practical value of bodily sensation mapping, a recently developed promising tool for emotional assessment. This tool, up to now, only captured sensations of activity/arousal (García-Magariño et al., 2018; Hietanen et al., 2016; Lyons et al., 2021; Nummenmaa et al., 2014; Sachs et al., 2019; Salazar-López et al., 2015; Torregrossa et al., 2018; Volynets et al., 2019), and we argued that further capturing sensations of valence-related bodily sensations is crucial for a comprehensive emotional assessment.

We showed that different emotional states are associated with topographically distinct sensations of valence-related bodily weight (lightness and heaviness). Participants reported feelings of bodily lightness in response to the positive emotions happiness, love, and pride. There was a particular increase in lightness in the upper body part (chest and head region) for happiness, possibly reflecting the “light-heartedness” associated with this emotion. Conversely, participants reported feelings of bodily heaviness for all negative emotions (anger, fear, disgust, anxiety, shame, envy, contempt, sadness, and depression). There was a particular peak in the chest region for sadness and depression, possibly reflecting the “heavy-heartedness” associated with these emotions. These patterns confirm that participants consistently expressed their specific embodied feelings of valence by means of BSMs, suggesting that valence-related bodily sensations are an essential part of the internal representation of emotions. Our results therefore provide further support for the circumplex model of affect, according to which valence is, besides activity, one of two fundamental and distinct dimensions of every emotional experience (Colibazzi et al., 2010; Lang et al., 1993; Posner et al., 2005, 2009). Specifically, we extended this two-dimensional approach used in the traditional emotional assessment to the format of body topography. Based on the activity BSMs, Nummenmaa et al. (2014) concluded that emotions are represented in the somatosensory system as categorical somatotopic maps. Our results suggest that there might be different somatosensory maps for the different dimensions of affect (e.g., activity and valence). In line with this, we found that the accuracy in recognizing emotions increased for both computers and humans (Experiment 2 and 3) when classification was based on activity- and valence-related BSMs compared to either type of BSM alone. This further confirms that both types of bodily sensations represent distinct parts of emotion knowledge, and that only the consideration of both activity and valence-related BSMs can capture the full range of emotional experience in the entire body (Colibazzi et al., 2010; Lang et al., 1993; Posner et al., 2005, 2009).

Besides establishing valence-related BSMs, a further aim of this study was to explore the possible dominance of one dimension of bodily sensations (activity or valence) for different emotions. Knowing which type of BSM is more informative might have practical relevance in case researchers or therapists are restricted to one bodily sensation (e.g., due to time-restricted assessments). We hypothesized that weight BSMs capture more clearly than activity BSMs the bodily sensations induced by emotions at the positive (happiness, love, pride) and negative (i.e., sadness, depression) endpoints of the valence spectrum, whereas we expected the opposite for emotions associated with the phylogenetically ancient fight or flight response (e.g., anger, fear). As a general remark, there was no difference in difficulty rating between weight and activity BSMs for all of these emotions. Thus, when instructed to do so, participants can equally well conceptualize their bodily sensations by means of weight or activity. This might further indicate that each emotion has an arousal and valence dimension, and that participants can flexibly rely on both (with the only exception of disgust). Nevertheless, it is still possible that one of those dimensions capture more clearly a specific emotional response when participants are forced to explicitly compare the two. This was the case for the clarity ratings, which showed several highly significant differences. The following discussion is based on the results from these explicit comparisons.

In line with our expectations, participants found it clearer to perceive and express their bodily sensations induced by sadness and depression in terms of bodily weight than in terms of bodily activity (Experiment 2), and sadness was better recognized based on the weight than on the activity BSMs (Experiment 2 and 3). In fact, the weight BSMs show a stringent pattern for emotions at the negative end of the valence spectrum, with a pronounced sensation of heaviness in the upper body part for sadness, and a more amplified and more widespread sensation of heaviness for depression. In contrast, the association between sensations of bodily activity and sadness and depression might be less clear. For activity BSMs, a sensation of activity was found in the upper body part, similar to the one also found in positive emotions such as happiness (see Nummenmaa et al., 2014 and also Fig. 3 of this study). It is somewhat paradoxical that two contradictory emotions (happiness and sadness) elicit a similar bodily sensation (activation in the chest). Moreover, unlike for sadness, there was no sensation of activation in the upper body part for depression. Given that sadness is a substantial marker of depression (Beck et al., 1996; Mouchet-Mages & Baylé, 2008), it is puzzling that bodily sensations can take opposite forms for these closely related emotions (activation for sadness and deactivation for depression). A possible explanation for these patterns might be that the dominant sensation in the upper body part for sadness might actually be a feeling of negative valence (i.e., heaviness), that is perceived as activation in the case of sadness, but is finally considered as deactivation when the feeling of heaviness becomes more intense in the case of depression. However that is, the case remains that activity BSMs show paradoxical patterns for sadness and depression (activation vs. deactivation, respectively), while weight BSMs show consistent patterns for sadness and depression (heaviness and still more heaviness, respectively). This points to a critical limitation of the previous (activity-only) BSM approach: since a sensation of activity seems ambiguous in terms of valence, the activity patterns alone can be difficult to interpret. The results from the clarity ratings suggest that the weight BSMs capture more accurately what participants actually feel and where they feel it when they are sad. To illustrate, the peaks in the chest and head regions of the weight BSM for sadness and depression is in accordance with what patients suffering from depression often report, for example in relation to antidepressant effects of ketamine treatment (“I had less *heaviness* in my throat and my chest”, or “All the pressure in my head just starts to feel *light* and normal again”) (Stocker et al., 2019). Given that depression is the leading disability worldwide affecting over 300 million people (World Health Organization, 2017), it is crucial to further advance tools that may help to better understand and assess sadness and depression, and this study sets the stage for the application of the newly conceptualized weight BSMs in a clinical sample.

In contrast to our hypothesis, there was no clear preference for bodily weight (vs. activity) in the clarity rating for the emotions at the positive end of the valence spectrum (happiness, love and pride), but these effects were not significant. Moreover, including weight BSMs did not improve performance when humans classified happiness and love, suggesting that a sensation of bodily lightness is not necessarily a more dominant (or salient) sensation than a sensation of bodily activation for these emotions. A possible explanation for this might be that happiness is—besides valence—also associated with approach motivation and therefore has a strong activation component (Colibazzi et al., 2010; Davidson, 1993; Davidson et al., 1990). It might also be possible that the pleasurable feeling of happiness is not unambiguously associated with a sensation of bodily lightness: a pleasant feeling has also been associated with heaviness, at least during deep relaxation (Smith, 2019).

In line with our hypothesis regarding activity BSMs, sensations induced by emotions associated with the phylogenetical ancient flight or fight response, such as anger and fear, were perceived and expressed more clearly in terms of activity than in terms of weight (Experiment 2). The same was true for disgust, surprise, envy, and contempt. Since the assumed function of anger and fear is to prepare the organism for an action (fight or flight), the primary bodily sensation might be related to these activating mechanisms (increase in heart rate, etc.). Disgust also leads to a cascade of activating mechanisms (e.g., wrinkling of nose, contraction of abdominals). Surprise, which is ambivalent in terms of valence, is often associated with either an approach or an avoidance behavior. It is therefore not surprising that activity might be the dominant bodily sensation for these emotions. In line with this, LDA classification performance for these emotions (anger, fear, disgust, surprise) was higher for activity than for weight BSMs, and also humans were more accurate when the classification of anger and disgust was based on activity BSMs rather than on weight BSMs. However, in contrast to the LDA, humans better recognized surprise based on weight than on activity BSMs. This might possibly be due to the following classification strategy: surprise was, besides the clearly positive emotions happiness, love, and pride, the only weight BSM with a positive valence (i.e., a sensation of lightness), which might have helped to recognize this emotion (all other remaining emotions had a negative valence). Nevertheless, there are some results for which we currently do not have an explanation, for example why humans recognized anxiety better based on the weight BSM than on the activity BSM in Experiment 3 (even though participants from Experiment 2 perceived anxiety more clearly in terms of bodily activity than in terms of bodily weight), and also why participants more clearly perceived envy and contempt in terms of bodily activity than in terms of bodily weight (Experiment 2). The latter might be related to findings showing that physiological responses (e.g., heart rate, cerebral activation) are generally stronger correlated with arousal than with valence (e.g., Keil et al., 2001; Lang et al., 1993). It should also be noted that envy and contempt were rated overall to be the most difficult ones to create BSMs, indicating that a body-specific tool might not be useful in assessing these two emotions. A possible reason for this might be that these two emotions are based on social context and deliberate appraisal: Both emotions arise in response to an unfavorable social comparisons; envy with others who are perceived as superior (Pila et al., 2016), and contempt with others who are perceived as inferior or worthless (Roseman, 2018). Thus, the exact function and physiological response pattern of these rather complex emotions might be less clear when compared to more basic emotions (e.g., Roseman, 2018). Moreover, expressing envy and contempt often provoke negative reactions in our social environment, and these emotions are therefore often regulated and suppressed, which may also suppress its physiological responses (Gross & Levenson, 1993). Furthermore, due to the social context, it might be more difficult to self-induce these emotions solely based on the emotion word in an isolated experimental setting. For these reasons, it might be more difficult to attribute specific bodily sensations to these emotions and create BSMs.

The finding of distinct weight-BSM patterns for different emotional states might be interpreted as support for discrete emotion theories according to which each emotion has a discrete physiological response patterns (Ekman, 1992; Izard, 1991; Tomkins, 1984). Nummenmaa et al. (2014) argued that the (activity) BSM patterns might reflect net sensations of different physiological systems (skeletomuscular and visceral sensations, and effects of autonomic nervous system). This might also be true for the weight BSMs, assuming that participants focus on the valence-related aspects of these various physiological systems. Accordingly, weight BSMs may reflect net sensations of physiological functions associated specifically with dorsal cortical areas and mesolimbic pathways (reward system), whereas activity BSMs might reflect net sensations of physiological functions associated specifically with midline and medial temporal lobe structures, since these neuronal networks have been associated with valence and activity, respectively (Colibazzi et al., 2010; Posner et al., 2009). William James first proposed that feelings are derived from sensing our bodily states (James, 1884), and it has been confirmed later that the mapping of varied features of bodily state in the central nervous system is a crucial requirement for the generation of feelings (e.g., Craig, 2002; Damasio, 1999; Montoya & Schandry, 1994; Wiens et al., 2000). The distinct somatotopic representation of valence might therefore be an important part of the generation of conscious feelings of emotions. However, it is important to note that the exact relationship between physiological changes and the reported bodily sensations in BSM assessments remains unclear (cf. Introduction). A combination of BSM assessment and whole-body physiological measurements (e.g., _15_O-H_2_O PET/MRT imaging) is required in the future to shed light into their exact relationship.

## Limitations and outlook

As a limitation of this study—as in all studies relying on subjective reports—we do not know to what extent BSMs reflect the actual sensations that participants had when putting themselves into the different emotional states, or whether also other strategies were recruited, such as activation of conceptual knowledge about the emotion and the bodily responses associated with it (i.e., sadness feels heavy in the chest). However, even if the BSMs were not entirely the result of bodily sensations induced by the experimental manipulations, it shows at least that activity and valence-related bodily sensations are both part of the conceptual knowledge of the emotion. Moreover, it is unlikely that specific bodily sensations would be part of conceptual knowledge without systematic experiences linking the concepts together (e.g., sadness and feeling of heaviness in the chest). According to the embodied view of cognition, the activation of conceptual knowledge about emotions involves simulation of bodily states and the reactivation of somatic responses (e.g., Barsalou, 2008; Dijkerman & Lenggenhager, 2018; Niedenthal et al., 2005b, 2009). It is therefore not clear whether it is at all possible to disentangle the different processes that contributed to the BSMs (e.g., direct experience of induced sensations, activation of conceptual knowledge, activation of a previous experience where this emotion was felt in a real-life situation). Importantly, the weight and activity BSMs for sadness induced by an emotion word from Experiment 1 and 2 closely resembled that of Experiment 4 in which sadness was induced by a sad movie scene. This suggests that bodily sensations induced by “reactivated” (or self-triggered) emotions are similar to those immediately induced by the stimulus. However, the preference for weight over activity BSMs was less pronounced when sadness was induced by a movie (Experiment 4) than by the emotion word (Experiment 2). This difference might be interpreted in favor of an influence of conceptual knowledge activation in response to the emotion word, but this difference could also be due to other methodological differences between these two experiments (e.g., different sample characteristics, group vs. single test situation, computerized vs. paper–pencil approach). In this sense, the methodological differences between Experiment 4 and 2 make it difficult to compare the results between the two emotion induction methods directly. Nummenmaa et al. (2014) also induced emotions by emotional movie scenes or guided emotional imagery, and they found the same BSM patterns as with emotion words, suggesting that semantics and stereotypes play only a minor role for BSMs. Future studies could assess the link between BSM patterns, interoceptive awareness/accuracy, and emotional responsiveness more systematically to further disentangle the sensory and cognitive mechanisms contributing to BSMs.

In this study, we used the concept of bodily weight as a proxy for valence. Besides bodily weight, other metaphors have also been related to emotions, such as body temperature (warm vs. cold), pressure, comfort/discomfort, tension, or enhancement (e.g., Barbosa Escobar et al., 2021; Bergman et al., 2015; Borhani et al., 2017; Kövecses, 1990; Rolls et al., 2008; Waggoner, 2010; Wilkowski et al., 2009). Thus, weight is only one form in which valence can be grounded in the body, and our choice of using bodily weight limits the generalizability of our findings. Future research should consider alternative ways to capture valence by BSMs to better understand which emotion is best captured by which concept, and whether similar BSMs can be found for different valence-related concepts. Regarding body temperature, it should be noted, however, that both positive and negative emotions (e.g., love, anger) have been associated with warmth (e.g., Kövecses, 2003; Lakoff & Kövecses, 1987; Wilkowski et al., 2009), suggesting that body temperature might not be a straightforward proxy for valence across different emotions.

Due to (1) the sample size, (2) the fact that our sample was not balanced for age and gender, and (3) the fact that our study did only include healthy participants, we were not able to further investigate individual differences and clinical signatures in weight BSMs. For the activity BSMs, it has already been shown that there are some moderate differences in BSMs between males and females (Volynets et al., 2019), between children and adults (Hietanen et al., 2016), and also some striking differences between non-clinical and clinical populations (depression: Lyons et al., 2021; autism: Palser et al., 2021; schizophrenia: Torregrossa et al., 2018). Future studies should extend this important line of research about individual differences and particularly about clinical implications also to valence-related BSMs.

Furthermore, differences between weight and activity BSMs were found only for clarity, but not for difficulty ratings. A possible explanation is that clarity might be more related to the question whether the concept (weight vs. activity) captures the quality of bodily sensations, while difficulty might be more related to the question how easy/difficult it is to allocate one’s bodily sensations onto the BSMs. Alternatively, it is possible that the direct comparison between the weight and activity concepts (in a forced-choice manner), as it was the case for the clarity ratings, is more sensitive to differences than when each concept is rated independently from each other, as it was the case for the difficulty rating. Due to the confound between the type of question (difficulty vs. clarity) and the experimental procedure (rating after each trial vs. comparative rating at the end), we are not able to disentangle these two possible sources with the present data.

Finally, in the human classification task (Experiment 3), participants allocated BSMs to emotion words in a forced-choice setting. This approach might have induced specific task strategies, as discussed in the Results and discussion section of Experiment 3. Such problems can be overcome by presenting each BSM separately and requiring participants to rate their level of agreement for each emotion. Such an approach might be used in future studies to better assess the certainty of classifications and more generally the extent to which emotions are represented as distinct categorical maps or as continuous/overlapping maps.

## Conclusions

In this study, we established for the first time valence-related BSMs that systematically capture bodily sensations associated with different emotions in a somatotopic format (lightness for positive emotions, heaviness for negative emotions). We also showed that emotions can be best recognized by humans and computers when classification is based on both activity and valence-related BSMs compared to either type of BSM alone, suggesting that both types of bodily sensations reflect distinct part of emotion knowledge. This finding is in line with the circumplex model of affect, according to which valence and arousal are two fundamental and distinct dimensions of every emotional experience (Colibazzi et al., 2010; Lang et al., 1993; Posner et al., 2005, 2009). Our study highlights that BSMs provide a fine-grained assessment of emotions by taking specific bodily sensations of valence and arousal into account, and therefore extend the traditional dimensional approaches in which typically only one global value (e.g., activity or valence rating on a scale) is considered. Participants found it rather easy to express their bodily sensations by means of BSMs (except for envy and contempt), suggesting that BSMs might be a powerful tool in assessing and communicating emotional feelings. Importantly, our results suggest that valence-related BSMs might be particularly important for the assessment of sadness and depression, possibly capturing more accurately what people feel when they are sad.

## Supplementary Information

Below is the link to the electronic supplementary material.

Supplementary file1 (DOCX 17345 KB)

## Data Availability

All data and supplemental material from this study can be found in the open science framework (OSF): https://osf.io/srvhp/. Additional material is available from the authors upon request.

## Footnotes

1 To illustrate, a Google search (8. June 2020) for the expressions “my chest feels negative”, “my chest feels unpleasant”, “my head feels pleasant”, “my head feels positive” yielded a total of two hits, while the same expressions with the terms heavy or light yielded more than 20′000 hits.

2 To compare our results to the original study by Nummenmaa et al. (2012), we followed their definition of basic emotions (anger, fear, disgust, sadness, happiness, and surprise) and non-basic emotions (anxiety, depression, pride, love, envy, contempt, shame). Note that the list of basic emotions is based on Ekman et al. (1969) from their research about the universality of facial expressions. Although alternative suggestions have been made (e.g., Plutchik, 2001; Tracy & Randles, 2011), these emotions are still the most commonly accepted candidates for basic emotions (Ekman, 2016; Kowalska & Wróbel, 2017).

## Code availability

Code will be available by the authors upon request.

## References

[CR1] Adolphs R (2017). How should neuroscience study emotions? By distinguishing emotion states, concepts, and experiences. Social Cognitive and Affective Neuroscience.

[CR2] Barbosa Escobar F, Velasco C, Motoki K, Byrne DV, Wang QJ (2021). The temperature of emotions. PLoS ONE.

[CR3] Barkow K, Heun R, Üstün TB, Berger M, Bermejo I, Gaebel W, Härter M, Schneider F, Stieglitz R-D, Maier W (2004). Identification of somatic and anxiety symptoms which contribute to the detection of depression in primary health care. European Psychiatry.

[CR4] Barrett LF (2006). Are Emotions natural kinds?. Perspectives on Psychological Science.

[CR5] Barrett LF, Bar M (2009). See it with feeling: Affective predictions during object perception. Philosophical Transactions of the Royal Society B: Biological Sciences.

[CR6] Barrett LF, Mesquita B, Ochsner KN, Gross JJ (2007). The experience of emotion. Annual Review of Psychology.

[CR7] Barrett LF, Wager TD (2006). The structure of emotion: Evidence from neuroimaging studies. Current Directions in Psychological Science.

[CR8] Barsalou LW (2008). Grounded cognition. Annual Review of Psychology.

[CR9] Beck AT, Steer RA, Brown GK (1996). Beck Depression Inventory-II. San Antonio.

[CR10] Becker-Weidman A, Hughes D (2008). Dyadic developmental psychotherapy: An evidence-based treatment for children with complex trauma and disorders of attachment. Child & Family Social Work.

[CR11] Bergman P, Ho H-N, Koizumi A, Tajadura-Jiménez A, Kitagawa N (2015). The pleasant heat? Evidence for thermal-emotional implicit associations occurring with semantic and physical thermal stimulation. Cognitive Neuroscience.

[CR12] Bliss-Moreau E, Williams LA, Santistevan AC (2020). The immutability of valence and arousal in the foundation of emotion. Emotion.

[CR13] Borghi, A. M., & Binkofski, F. (2014). *Words as Social Tools: An Embodied View on Abstract Concepts*. Springer. 10.1007/978-1-4614-9539-0

[CR14] Borhani K, Làdavas E, Fotopoulou A, Haggard P (2017). “Lacking warmth”: Alexithymia trait is related to warm-specific thermal somatosensory processing. Biological Psychology.

[CR15] Bradley MM, Lang PJ (1994). Measuring emotion: The Self-Assessment Manikin and the semantic differential. Journal of Behavior Therapy and Experimental Psychiatry.

[CR16] Breuning, L. G. (2016). *Habits of a Happy Brain: Retrain Your Brain to Boost Your Serotonin, Dopamine, Oxytocin, & Endorphin Levels*. Adams Media.

[CR17] Broekens J, Brinkman W-P (2013). AffectButton: A method for reliable and valid affective self-report. International Journal of Human-Computer Studies.

[CR18] Brownlee, J. (2021, January). Machine learning mastery. *Repeated K-Fold Cross-Validation for Model Evaluation in Python*. https://machinelearningmastery.com/repeated-k-fold-cross-validation-with-python/

[CR19] Buck, R. (1985). Prime Theory: An Integrated View of Motivation and Emotion. *Psychological Review*, *92*(3), 389–413. insights.ovid.com

[CR20] Bush LE (1973). Individual differences multidimensional scaling of adjectives denoting feelings. Journal of Personality and Social Psychology.

[CR21] Cacioppo JT, Berntson GG (1994). Relationship between attitudes and evaluative space: A critical review, with emphasis on the separability of positive and negative substrates. Psychological Bulletin.

[CR22] Cacioppo, J. T., Berntson, G. G., Larson, J., & T., Poehlmann, K. M., & Ito, T. A. (2000). The psychophysiology of emotion. *Handbook of Emotions* (pp. 173–191). Guilford Press.

[CR23] Cassavetes, N. (2009). *My sister’s keeper [Beim Leben meiner Schwester]* [Drama; Movie]. Warner Bros.

[CR24] Cavanagh SR, Glode RJ, Opitz PC (2015). Lost or fond? Effects of nostalgia on sad mood recovery vary by attachment insecurity. Frontiers in Psychology.

[CR25] Colibazzi T, Posner J, Wang Z, Gorman D, Gerber A, Yu S, Zhu H, Kangarlu A, Duan Y, Russell JA, Peterson BS (2010). Neural systems subserving valence and arousal during the experience of induced emotions. Emotion.

[CR26] Craig AD (2002). How do you feel? Interoception: The sense of the physiological condition of the body. Nature Reviews Neuroscience.

[CR27] Damasio, A. (1999). *The Feeling of What Happens: Body and Emotion in the Making of Consciousness* (p. 386). Harcourt College Publishers.

[CR28] Damasio, A. (2004). *Emotions and Feelings*. Cambridge University Press.

[CR29] Damasio A, Carvalho GB (2013). The nature of feelings: Evolutionary and neurobiological origins. Nature Reviews Neuroscience.

[CR30] Damjanovic L, Santiago J (2016). Contrasting vertical and horizontal representations of affect in emotional visual search. Psychonomic Bulletin & Review.

[CR31] Davidson RJ (1993). Parsing affective space: Perspectives from neuropsychology and psychophysiology. Neuropsychology.

[CR32] Davidson RJ, Ekman P, Saron CD, Senulis JA, Friesen WV (1990). Approach-withdrawal and cerebral asymmetry: Emotional expression and brain physiology: I. Journal of Personality and Social Psychology.

[CR33] Davitz, J. R. (2016). *The Language of Emotion*. Academic Press.

[CR34] Dijkerman C, Lenggenhager B (2018). The body and cognition: The relation between body representations and higher level cognitive and social processes. Cortex.

[CR35] Ekman P (1992). An argument for basic emotions. Cognition and Emotion.

[CR36] Ekman P (2016). What scientists who study emotion agree about. Perspectives on Psychological Science.

[CR37] Ekman P, Friesen WV (1971). Constants across cultures in the face and emotion. Journal of Personality and Social Psychology.

[CR38] Ekman P, Levenson RW, Friesen WV (1983). Autonomic nervous system activity distinguishes among emotions. Science.

[CR39] Ekman P, Sorenson ER, Friesen WV (1969). Pan-cultural elements in facial displays of emotion. Science.

[CR40] Farrell PA (1985). Exercise and endorphins-male responses. Medicine and Science in Sport and Exercies.

[CR41] Faul F, Erdfelder E, Lang A-G, Buchner A (2007). G*Power 3: A flexible statistical power analysis program for the social, behavioral, and biomedical sciences. Behavior Research Methods.

[CR42] Foglia L, Wilson RA (2013). Embodied cognition. *WIREs*. Cognitive Science.

[CR43] Forgas JP (1994). The role of emotion in social judgments: An introductory review and an Affect Infusion Model (AIM). European Journal of Social Psychology.

[CR44] Frijda NH (1987). Emotion, cognitive structure, and action tendency. Cognition and Emotion.

[CR45] Fuchs T, Schlimme JE (2009). Embodiment and psychopathology: A phenomenological perspective. Current Opinion in Psychiatry.

[CR46] Gainotti, G. (2020). What Are Emotions. In G. Gainotti (Ed.), *Emotions and the Right Side of the Brain* (pp. 3–11). Springer International Publishing. 10.1007/978-3-030-34090-2_2

[CR47] García-Magariño I, Chittaro L, Plaza I (2018). Bodily sensation maps: Exploring a new direction for detecting emotions from user self-reported data. International Journal of Human-Computer Studies.

[CR48] Gigerenzer, G., & Goldstein, D. G. (1999). Betting on one good reason: The take the best heuristic. *Simple heuristics that make us smart* (pp. 75–95). Oxford University Press.

[CR49] Gomez P, Zimmermann P, Guttormsen-Schär S, Danuser B (2005). Respiratory responses associated with affective processing of film stimuli. Biological Psychology.

[CR50] Gross JJ, Levenson RW (1993). Emotional suppression: Physiology, self-report, and expressive behavior. Journal of Personality and Social Psychology.

[CR51] Haidt J, McCauley C, Rozin P (1994). Individual differences in sensitivity to disgust: A scale sampling seven domains of disgust elicitors. Personality and Individual Differences.

[CR52] Hamdi S (2016). A cognitive study of happiness metaphors in English, Tunisian Arabic and Spanish. Arab World English Journal (AWEJ).

[CR53] Harmon-Jones C, Bastian B, Harmon-Jones E (2016). The discrete emotions questionnaire: A new tool for measuring state self-reported emotions. PLoS ONE.

[CR54] Harmon-Jones E, Harmon-Jones C, Abramson L, Peterson CK (2009). PANAS positive activation is associated with anger. Emotion.

[CR55] Harmon-Jones E, Harmon-Jones C, Summerell E (2017). On the importance of both dimensional and discrete models of emotion. Behavioral Sciences.

[CR56] Hietanen JK, Glerean E, Hari R, Nummenmaa L (2016). Bodily maps of emotions across child development. Developmental Science.

[CR57] Hufendiek, R. (2015). *Embodied Emotions: A Naturalist Approach to a Normative Phenomenon*. Routledge.

[CR58] Hung Y, Zheng X, Carlson J, Giurge LM (2017). The weight of the saddened soul: The bidirectionality between physical heaviness and sadness and its implications for sensory marketing. Journal of Marketing Management.

[CR59] Izard, C. E. (2013). *Human Emotions*. Springer Science & Business Media.

[CR60] Izard, C. E. (1991). *The Psychology of Emotions*. Plenum Press.

[CR61] Izard CE (2007). Basic emotions, natural kinds, emotion schemas, and a new paradigm. Perspectives on Psychological Science.

[CR62] James W (1884). What is an emotion?. Mind.

[CR63] Johnson M (2015). Embodied understanding. Frontiers in Psychology.

[CR64] Jung W-M, Ryu Y, Lee Y-S, Wallraven C, Chae Y (2017). Role of interoceptive accuracy in topographical changes in emotion-induced bodily sensations. PLoS ONE.

[CR65] Keil A, Müller MM, Gruber T, Wienbruch C, Stolarova M, Elbert T (2001). Effects of emotional arousal in the cerebral hemispheres: A study of oscillatory brain activity and event-related potentials. Clinical Neurophysiology.

[CR66] Kim J, André E (2008). Emotion recognition based on physiological changes in music listening. IEEE Transactions on Pattern Analysis and Machine Intelligence.

[CR67] Konishi N, Himichi T, Ohtsubo Y (2019). Heart rate reveals the difference between disgust and anger in the domain of morality. Evolutionary Behavioral Sciences.

[CR68] Kövecses, Z. (1990). *Emotion concepts*. Springer. 10.1007/978-1-4612-3312-1

[CR69] Kövecses, Z. (2003). *Metaphor and Emotion: Language, Culture, and Body in Human Feeling*. Cambridge University Press.

[CR70] Kowalska, M., & Wróbel, M. (2017). Basic Emotions. In V. Zeigler-Hill & T. K. Shackelford (Eds.), *Encyclopedia of Personality and Individual Differences* (pp. 1–6). Springer International Publishing. 10.1007/978-3-319-28099-8_495-1

[CR71] Kragel PA, LaBar KS (2016). Decoding the nature of emotion in the brain. Trends in Cognitive Sciences.

[CR72] Kuhn, M. (2008). Building Predictive Models in R Using the caret Package. *Journal of Statistical Software*, *028*(i05). https://ideas.repec.org/a/jss/jstsof/v028i05.html

[CR73] Lakoff G, Johnson M (1980). Conceptual metaphor in everyday language. The Journal of Philosophy.

[CR74] Lakoff G, Kövecses Z (1987). The cognitive model of anger inherent in American English. Cultural Models in Language and Thought.

[CR75] Lang PJ, Bradley MM, Cuthbert BN (1998). Emotion, motivation, and anxiety: Brain mechanisms and psychophysiology. Biological Psychiatry.

[CR76] Lang PJ, Greenwald MK, Bradley MM, Hamm AO (1993). Looking at pictures: Affective, facial, visceral, and behavioral reactions. Psychophysiology.

[CR77] Lench HC, Flores SA, Bench SW (2011). Discrete emotions predict changes in cognition, judgment, experience, behavior, and physiology: A meta-analysis of experimental emotion elicitations. Psychological Bulletin.

[CR78] Levenson, R. W. (2003). Blood, Sweat, and Fears: The Autonomic Architecture of Emotion. *Annals of the New York Academy of Sciences*, *1000*, 348–366. insights.ovid.com 10.1196/annals.1280.016 14766648

[CR79] Levenson RW (2014). The autonomic nervous system and emotion. Emotion Review.

[CR80] Levenson RW (2019). Reflections on 30 years of Cognition & Emotion. Cognition and Emotion.

[CR81] *LimeSurvey: An open source survey tool*. (2012). LimeSurvey GmbH. http://www.limesurvey.org

[CR82] Lyons N, Strasser A, Beitz B, Teismann T, Ostermann T, Anderle L, Michalak J (2021). Bodily maps of emotion in major depressive disorder. Cognitive Therapy and Research.

[CR83] Lyubomirsky S, Lepper HS (1999). A measure of subjective happiness: Preliminary reliability and construct validation. Social Indicators Research.

[CR84] McNair, D. M., Lorr, M., & Droppelman, L. F. (1971). *Manual for the Profile of Mood States*. Educational and Industrial Testing Services.

[CR85] Mehrabian A (1995). Framework for a comprehensive description and measurement of emotional states. Genetic, Social, and General Psychology Monographs.

[CR86] Mehrabian A (1996). Pleasure-arousal-dominance: A general framework for describing and measuring individual differences in Temperament. Current Psychology.

[CR87] Montoya P, Schandry R (1994). Emotional experience and heartbeat perception in patients with spinal cord injury and control subjects. Journal of Psychophysiology.

[CR88] Moradi MR, Mashak SP (2013). A comparative and contrastive study of sadness conceptualization in Persian and English. English Linguistics Research.

[CR89] Mouchet-Mages S, Baylé FJ (2008). Sadness as an integral part of depression. Dialogues in Clinical Neuroscience.

[CR90] Nakul E, Dabard C, Toupet M, Hautefort C, van Nechel C, Lenggenhager B, Lopez C (2020). Body-maps of emotions in bilateral vestibulopathy. Journal of Neurology.

[CR91] Nardelli M, Valenza G, Greco A, Lanata A, Scilingo EP (2015). Recognizing emotions induced by affective sounds through heart rate variability. IEEE Transactions on Affective Computing.

[CR92] Niedenthal PM, Barsalou LW, Winkielman P, Krauth-Gruber S, Ric F (2005). Embodiment in attitudes, social perception, and emotion. Personality and Social Psychology Review.

[CR93] Niedenthal PM, Winkielman P, Mondillon L, Vermeulen N (2009). Embodiment of emotion concepts. Journal of Personality and Social Psychology.

[CR94] Nummenmaa L, Glerean E, Hari R, Hietanen JK (2014). Bodily maps of emotions. Proceedings of the National Academy of Sciences.

[CR95] Oatley K, Johnson-laird PN (1987). Towards a cognitive theory of emotions. Cognition and Emotion.

[CR96] Osgood, C. E., Suci, G. J., & Tannenbaum, P. H. (1957). *The Measurement of Meaning*. University of Illinois Press.

[CR97] Palser ER, Galvez-Pol A, Palmer CE, Hannah R, Fotopoulou A, Pellicano E, Kilner JM (2021). Reduced differentiation of emotion-associated bodily sensations in autism. Autism.

[CR98] Panksepp J (2007). Neurologizing the psychology of affects: How appraisal-based constructivism and basic emotion theory can coexist. Perspectives on Psychological Science.

[CR99] Pila E, Brunet J, Crocker PRE, Kowalski KC, Sabiston CM (2016). Intrapersonal characteristics of body-related guilt, shame, pride, and envy in Canadian adults. Body Image.

[CR100] Plutchik, R. (2001). The Nature of Emotions: Human emotions have deep evolutionary roots, a fact that may explain their complexity and provide tools for clinical practice. *American Scientist*, *89*(4), 344–350. JSTOR. https://www.jstor.org/stable/27857503

[CR101] Posner J, Russell JA, Gerber A, Gorman D, Colibazzi T, Yu S, Wang Z, Kangarlu A, Zhu H, Peterson BS (2009). The neurophysiological bases of emotion: An fMRI study of the affective circumplex using emotion-denoting words. Human Brain Mapping.

[CR102] Posner J, Russell JA, Peterson BS (2005). The circumplex model of affect: An integrative approach to affective neuroscience, cognitive development, and psychopathology. Development and Psychopathology.

[CR103] Rimé B, Philippot P, Cisamolo D (1990). Social schemata of peripheral changes in emotion. Journal of Personality and Social Psychology.

[CR104] Rolls ET, Grabenhorst F, Parris BA (2008). Warm pleasant feelings in the brain. NeuroImage.

[CR105] Roseman, I. J. (2018). Rejecting the unworthy: The causes, components, and consequences of contempt. In M. Mason (Ed.), *The moral psychology of contempt* (pp. 107–130). Rowman & Littlefield.

[CR106] Russel JA (1980). A circumplex model of affect. Journal of Personality and Social Psychology.

[CR107] Russell JA (2003). Core affect and the psychological construction of emotion. Psychological Review.

[CR108] Saarni, C. (1999). *The Development of Emotional Competence*. Guilford Press.

[CR109] Sachs ME, Kaplan J, Habibi A (2019). Echoing the emotions of others: Empathy is related to how adults and children map emotion onto the body. Cognition and Emotion.

[CR110] Salazar-López E, Domínguez E, Ramos VJ, De la Fuente J, Meins A, Iborra O, Gálvez G, Rodríguez-Artacho MA, Gómez-Milán E (2015). The mental and subjective skin: Emotion, empathy, feelings and thermography. Consciousness and Cognition.

[CR111] Scherer, K. (2000). *Psychological Models of Emotion*. Oxford University Press.

[CR112] Schlosberg H (1954). Three dimensions of emotion. Psychological Review.

[CR113] Schwarz, N. (1990). Feelings as information: Informational and motivational functions of affective states. *Handbook of Motivation and Cognition: Foundations of Social Behavior* (pp. 527–561). The Guilford Press.

[CR114] Scrimin S, Mason L (2015). Does mood influence text processing and comprehension? Evidence from an eye-movement study. British Journal of Educational Psychology.

[CR115] Shaver P, Schwartz J, Kirson D, O’Connor C (1987). Emotion knowledge: Further exploration of a prototype approach. Journal of Personality and Social Psychology.

[CR116] Siegel E, Sands M, den Noortgate WV, Condon P, Chang Y, Dy J, Quigley K, Barrett L (2018). Emotion fingerprints or emotion populations? A meta-analytic investigation of autonomic features of emotion categories. Psychological Bulletin.

[CR117] Smith, J. C. (2019). *Third-Generation Mindfulness AND The Universe of Relaxation: Professional Version* (1st ed.). Kendall Hunt Publishing.

[CR118] Smith RH, Parrott WG, Diener EF, Hoyle RH, Kim SH (1999). Dispositional envy. Personality and Social Psychology Bulletin.

[CR119] Spielberger, C. D. (2010). *State-Trait Anxiety Inventory. In The Corsini Encyclopedia of Psychology* (p. 1). American Cancer Society. 10.1002/9780470479216.corpsy0943

[CR120] Stocker K, Hasler G, Hartmann M (2019). The altered-state-of-consciousness (ASC) aspect of a feeling of lightness is reported to be associated with antidepressant benefits by depressed individuals receiving ketamine infusions: A systematic analysis of internet video testimonials. Psychotherapy and Psychosomatics.

[CR121] Tajadura-Jiménez, A., Basia, M., Deroy, O., Fairhurst, M., Marquardt, N., & Bianchi-Berthouze, N. (2015). As light as your footsteps: Altering walking sounds to change perceived body weight, emotional state and gait. *Proceedings of the 33rd Annual ACM Conference on Human Factors in Computing Systems*, 2943–2952. 10.1145/2702123.2702374

[CR122] Tebeka S, Pignon B, Amad A, Le Strat Y, Brichant-Petitjean C, Thomas P, Vaiva G, Roelandt J-L, Benradia I, Etain B, Rolland B, Dubertret C, Geoffroy PA (2018). A study in the general population about sadness to disentangle the continuum from well-being to depressive disorders. Journal of Affective Disorders.

[CR123] Tomkins, S. S. (1984). Affect theory. *Approaches To Emotion* (pp. 163–195). Psychology Press.

[CR124] Torregrossa LJ, Snodgress MA, Hong SJ, Nichols HS, Glerean E, Nummenmaa L, Park S (2018). Anomalous Bodily Maps of Emotions in Schizophrenia. Schizophrenia Bulletin..

[CR125] Tracy JL, Randles D (2011). Four models of basic emotions: A review of Ekman and Cordaro, Izard, Levenson, and Panksepp and Watt. Emotion Review.

[CR126] Tracy JL, Robins RW (2007). The psychological structure of pride: A tale of two facets. Journal of Personality and Social Psychology.

[CR127] van Schalkwyk GI, Wilkinson ST, Davidson L, Silverman WK, Sanacora G (2018). Acute psychoactive effects of intravenous ketamine during treatment of mood disorders: Analysis of the clinician administered dissociative state scale. Journal of Affective Disorders.

[CR128] Volynets S, Glerean E, Hietanen JK, Hari R, Nummenmaa L (2019). Bodily maps of emotions are culturally universal. Emotion.

[CR129] Waggoner JE (2010). Temperature-based metonymies for emotions in children and adults. Psychological Reports.

[CR130] Watson DC, Clark LA, Tellegen A (1988). Development and validation of brief measures of positive and negative affect: The PANAS scales. Journal of Personality and Social Psychology.

[CR131] Wenfeng L (2008). Chou (Sadness) in Chinese and English writings: an experimental study/CHOU (TRISTESSE) DANS LES OUVRAGES CHINOIS ET ANGLAIS: UNE ÉTUDE EXPÉRIMENTALE. Cross-Cultural Communication.

[CR132] Wiens S (2005). Interoception in emotional experience. Current Opinion in Neurology.

[CR133] Wiens S, Mezzacappa ES, Katkin ES (2000). Heartbeat detection and the experience of emotions. Cognition and Emotion.

[CR134] Wilkowski BM, Meier BP, Robinson MD, Carter MS, Feltman R (2009). “Hot-headed” is more than an expression: The embodied representation of anger in terms of heat. Emotion.

[CR135] World Health Organization. (2017). *Depression and Other Common Mental Disorders: Global Health Estimates*. World Health Organization.

[CR136] Wundt, W. (1905). *Grundzüge der physiologischen Psychologie [Fundamentals of physiological psychology]* (5th ed.). Engelmann.

[CR137] Yu N (1995). Metaphorical expressions of anger and happiness in English and Chinese. Metaphor and Symbol.

[CR138] Zhao X, He X, Zhang W (2016). A heavy heart: The association between weight and emotional words. Frontiers in Psychology.

[CR139] Zuckerman M, Lubin B, Robins S (1965). Validation of the multiple affect adjective check list in clinical situations. Journal of Consulting Psychology.

