# Supplementary material for: Happiness feels light and sadness feels heavy: introducing valence-related bodily sensation maps of emotions
Source: Psychol Res. 2022 Feb 28;87(1):59–83. doi: 10.1007/s00426-022-01661-3 (PMC9873729; doi:10.1007/s00426-022-01661-3)
Supplement: Supplementary file 1 — Supplementary file1 (DOCX 17345 KB) [file 426_2022_1661_MOESM1_ESM.docx]

**Happiness feels light, sadness feels heavy: introducing valence-related bodily sensation maps of emotions**

Supplemental material – Figure S1-S4

Experiment 2

| Anger | Fear | Disgust | | Happiness | | Sadness | Surprise | Neutral |  |
| --- | --- | --- | --- | --- | --- | --- | --- | --- | --- |
|  | | | | | | | | | Lightness |
|  |  |  |  |  |  |  |  |  |  |
| Anxiety | Love | Depression | Contempt | | Pride | | Shame | Envy | Heaviness |
|  | | | | | | | | |  |

| Anger | Fear | Disgust | | Happiness | | Sadness | Surprise | Neutral |  |
| --- | --- | --- | --- | --- | --- | --- | --- | --- | --- |
|  | | | | | | | | | Activation |
|  |  |  |  |  |  |  |  |  |  |
| Anxiety | Love | Depression | Contempt | | Pride | | Shame | Envy | De-activation |
|  | | | | | | | | |  |

*Figure S1.* Full list of statistical weight (upper half) and activity (lower half) BSMs created in Experiment 2.

| Basic emotions (and the neutral state) | |
| --- | --- |
| 1. Weight-BSMs | 1. Activity-BSMs |
|  |  |
| 1. Weight- and activity BSMs | |
|  | |

*Figure S2*. Classification matrices of the linear discriminant analysis from Experiment 2 for the basic emotions and the neural state when classification was based on weight-BSMs (a.), activity-BSMs (b.), or both BSMs (c.). Emotions along the horizontal axis reflect the true, and those along the vertical axis reflect the classified emotions (the values in each column sum up to 1). The chance level was 0.14 (1/7).

| All 13 emotions (and the neutral state) | |
| --- | --- |
| 1. Weight-BSMs | 1. Activity-BSMs |
|  |  |
| 1. Weight- and activity BSMs | |
|  | |

*Figure S3*. Classification matrices of the linear discriminant analysis from Experiment 2 for the basic emotions and the neural state when classification was based on weight-BSMs (a.), activity-BSMs (b.), or both BSMs (c.). Emotions along the horizontal axis reflect the true, and those along the vertical axis reflect the classified emotions (the values in each column sum up to 1). The chance level was 0.07 (1/14).

Experiment 3: Human classification accuracy

|  |
| --- |
|  |
|  |

*Figure S4*. Layout of the online-emotion categorization task in Experiment 3. The upper part (a.) shows the first categorization task with only one type of BSMs (in this example activity-BSMs), and the lower part (b.) shows the second categorization task with both BSMs (activity BSMs on the left, and weight-BSMs on the right of the central number).
